# Advances in Natural Products from Mangrove-Associated Fungi Along the Indian Ocean Coast

**DOI:** 10.3390/molecules31020261

**Published:** 2026-01-12

**Authors:** Parakkrama Wijerathna, Xinqi Chen, Rongxiang Qiu, P.V.J.S. Wijethilake, Yi Chen, Nuwan Madushanka, I.J.J.U.N. Perera, Jian Cai, Lalith Jayasinghe, Yonghong Liu, Vajira P. Bulugahapitiya, Xuefeng Zhou

**Affiliations:** 1 State Key Laboratory of Tropical Oceanography, South China Sea Institute of Oceanology, Chinese Academy of Sciences, Guangzhou 510301, China; parabmpd@gmail.com (P.W.); chenxinqi24@mails.ucas.ac.cn (X.C.); 2112450093@stu.gdpu.edu.cn (R.Q.); chenyi221@mails.ucas.ac.cn (Y.C.); caijian@scsio.ac.cn (J.C.); 2 University of Chinese Academy of Sciences, Beijing 100049, China; 3 School of Chinese Materia Medica, Guangdong Pharmaceutical University, Guangzhou 510006, China; 4 Department of Agricultural Biology, Faculty of Agriculture, Eastern University, Chenkalady 30350, Sri Lankanuwanmadushanka404@gmail.com (N.M.); 5 National Institute of Fundamental Studies, Hantana Road, Kandy 200000, Sri Lanka; lalith.ja@nifs.ac.lk; 6 Department of Chemistry, Faculty of Science, University of Ruhuna, Matara 81000, Sri Lanka; 7 The China–Sri Lanka Joint Center for Education and Research, South China Sea Institute of Oceanology, Chinese Academy of Sciences, Guangzhou 510301, China

**Keywords:** chemical diversity, Indian Ocean coast, mangrove-associated fungi, bioactive secondary metabolites, drug discovery

## Abstract

Mangrove ecosystems along the Indian Ocean coast show great biodiversity, adapting to harsh environmental conditions of high salinity and higher organic matter, and they are a host for a range of microbial communities with special features that produce unique secondary metabolites. Of this, mangrove-associated endophytic fungi, the second largest ecological group of marine fungi, show the greater potential, being a diverse pool for discovering novel bio-actives with pharmacological and biotechnological interest. This review summarizes the research findings on structural diversity and the associated pharmacological activities of secondary metabolites produced by mangrove-associated fungi along the Indian Ocean coast reported over the period of 2002–2025, based on the literature retrieved from Google Scholar. The total of **302** secondary metabolites is presented mainly from classes of polyketides (**208**), alkaloids (**34**), and terpenoids (**60**). Interestingly, **164** compounds were identified, as first reported in those publications. These compounds have been reported to show diverse biological activities, and the most prominent activities are cytotoxic, antibacterial, antifungal, antioxidant, enzyme inhibitory, and anti-inflammatory effects. The structural novelty and pharmacological activities of these metabolites highlight the importance of mangrove fungi as promising sources for new drug discovery and advancing industrial biotechnology. Therefore, this review highlights the insight into the possible application of these chemical compounds in the future drug industry, as well as in biotechnology for advancing human well-being. Furthermore, though significant progress has been made in exploring the fungi community from mangroves of the African and Middle Eastern coasts, the Indian coast mangrove fungi are yet to be explored more for novel discoveries.

## 1. Introduction

Marine fungi, which have a habitat in mangrove ecosystems, are widely distributed on the Indian Ocean coast and are recognized as an important source of natural products due to their biosynthetic process of important secondary metabolites [1]. It is documented that about 625 fungal species come from mangroves, which represents only about 0.62% of the fungal species described in the world [2].

The mangrove forests constitute highly complex ecosystems that occur in tropical and subtropical intertidal estuarine zones and host diverse microorganisms, including endophytic fungi, actinomycetes, bacteria, cyanobacteria, algae, and protozoa. In the tropical mangrove microbial community, bacteria and fungi account for 91% of the total microbial biomass [3,4]. These mangrove-associated fungi represent the second-largest ecological group of marine fungi [1]. These fungi, which thrive in mangrove ecosystems, have evolved to withstand frequent and unpredictable environmental changes, such as high salinity, low oxygen levels, limited nutrient availability, tidal fluctuations, elevated temperatures, intense light exposure, and droughts [5]. They not only play a vital role in shaping and supporting the mangrove biosphere but also serve as an efficient mechanism for metabolic pathways in fungi, which has led to the production of a structurally unique and diverse pool of bioactive secondary metabolites [6].

### Diversity of Mangroves Along the Indian Ocean Coast and Diversity of Endophytic Fungi Associated

Mangroves are a drastic, environmentally tolerant ecosystem found mainly in tropical and sub-tropical intertidal zones around the world. The Indian Ocean has been widely recognized as one of the key hubs for harboring more than 70 mangrove species across about 30 countries [7]. As reported by [7], mangroves in the Indian Ocean occupy approximately 85,000 km^2^, accounting for nearly 47% of the world’s total mangrove area (estimated at 18 million hectares). In the Asian region bordered by the Indian Ocean, Indonesia dominates the spatial coverage of mangroves (32,500 km^2^) [8], followed by Myanmar (6287 km^2^ [9], Malaysia (5800 km^2^) [10], India (4975 km^2^) [11], and Bangladesh (4950 km^2^) [12]. Despite the dominant Asian region, countries in Africa, namely Mozambique, Tanzania, and Madagascar, show a spatial coverage of 2776 km^2^, 544.3 km^2^, and 2776 km^2^ [13], respectively.

A total of 55 mangrove species belong to 22 genera, and 18 families have been known from the Indian Ocean coast [14]. This covers 85% of the world’s total mangrove species, showing 65 species on average. Countries such as Indonesia (48), Malaysia (38), India (39), Thailand (33), and Myanmar (31) are rich in mangroves. *Avicennia marina*, *Rhizophora mucronata*, *Bruguiera* spp., *Ceriops tagal*, and *Sonneratia* spp. are among the most prominent mangrove species along the Indian Ocean coast. There are some endemic species to South-East Asia and Northern Australia on the Indian Ocean coast. More details are given in Table 1.

All these well-sustained floras under a drastic environment provide hosts for diverse endophytic fungi. These mangrove-hosted marine fungi synthesize specific secondary metabolites with important bioactivities and unique structural features, providing a compound pool for novel drug discoveries and other applications [1,37].

## 2. Structural Diversity and Pharmacological Activities of Secondary Metabolites Isolated from Mangrove-Associated Fungi of the Indian Coast

The exploration of bioactive lead compounds from mangrove endophytic fungi has emerged as a vibrant frontier within natural product chemistry research [38]. These endophytic fungi synthesize structurally diverse biologically active compounds [39] that support the mangrove plants within harsh environmental and soil conditions in a coastal environment [40]. Therefore, these specific activities of secondary metabolites can be used in various biotechnological, pharmacological, and industrial applications, such as phytohormone, bioremediation, biofertilization, biocontrol, immunosuppressive, antiparasitic, antimicrobial, antitumor, and antioxidant activity [2]. These bioactive metabolites are recognized as alkaloids, polyketides, and terpenoids, the main categories of natural products [1,6,41]. The following sections discuss these fungal metabolites based on their chemical structural classes, highlighting their structural diversity and diverse biological activities. Special consideration is given to the classes of polyketides, terpenoids, and alkaloids.

### 2.1. Polyketides

Polyketides are the most abundant group of fungal secondary metabolites, characterized by their remarkable structural and functional diversity [1,42]. These compounds are known to display a broad spectrum of biological activities, including antibacterial [6,37,40], antifungal [6,37,40,43,44], anticancer [45], antiviral [46], immunosuppressive [40], cholesterol-lowering [45], and anti-inflammatory effects [37,40]. Polyketides biosynthesized in mangrove-associated fungi can be categorized into seven main structural groups, and several subgroups belong [46]. The main groups are quinones, naphthalene, lactones, phenolic compounds, coumarins and chromone derivatives, xanthone, benzofuran, etc. Fungal polyketide compounds such as chromones, quinones, naphthalene, and phenols are known to exhibit significant potential in drug development and agricultural biocontrol due to their unique chemical structures and broad pharmacological activities [1].

In this review, we presented **208** polyketides identified from Indian Ocean coast mangrove-associated fungi. Among them, **100** were found to be first reported in these papers. Out of them all, about **104** compounds are reported to possess anti-microbial, cytotoxic, antioxidant, etc., effects. The details on the structural properties and biological activities of each category of polyketides are explained below.

#### 2.1.1. Quinones

**Figure 1:**
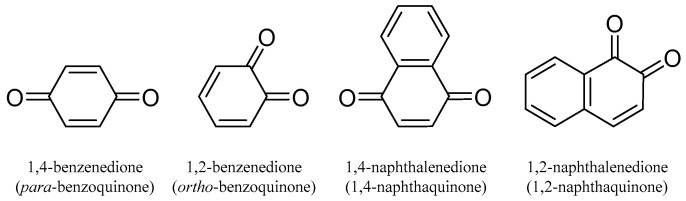
The basic chemical structures of quinones.

Quinones are conjugated cyclic diketones. The most common are 1,2 or 1,4-diketones. The basic structures are shown in Figure 1.

**Figure 2:**
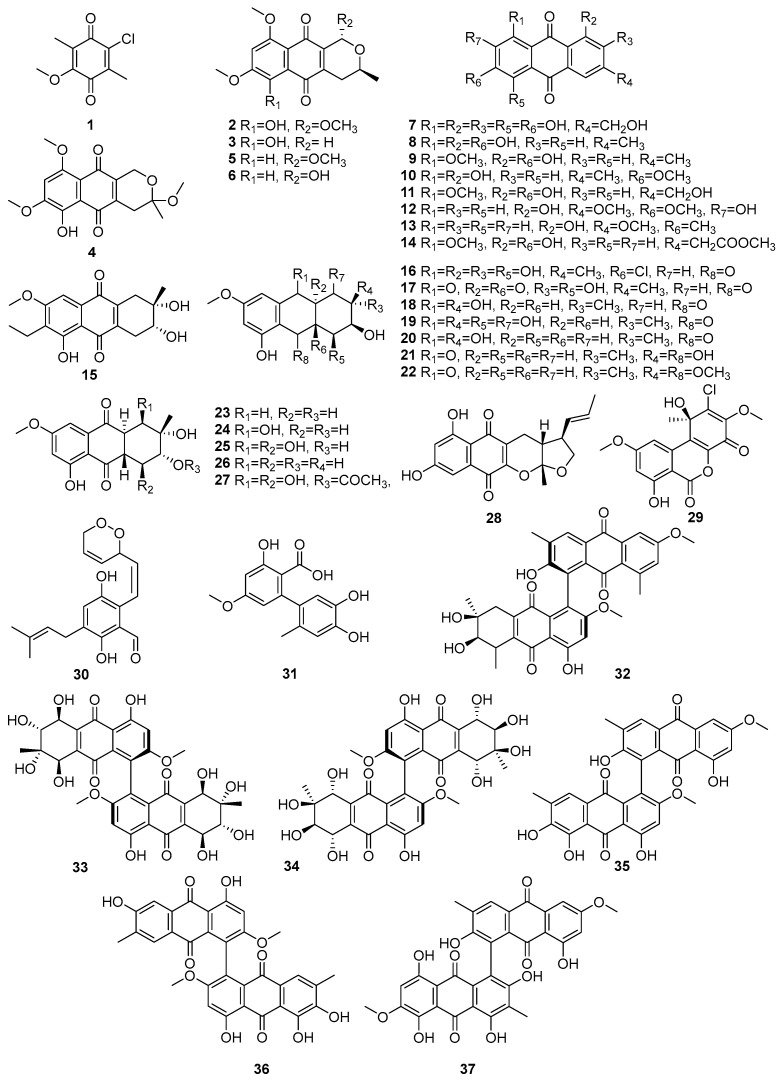
Quinones in polyketide compounds isolated from mangrove-associated fungi (**1**–**37**).

Fungi produce structurally diverse quinone metabolites, which are mainly polyketide-derived compounds. Figure 2 (**1**–**37**) displays the quinones in the polyketide compounds isolated from mangrove-associated fungi. A known compound, 2-chloro-5-methoxy-3-methylcyclohexa-2,5-diene-1,4-dione (**1**), was isolated from the mangrove-derived fungus *Xylaria cubensis* PSU-MA34 obtained from a branch of *Bruguiera parviflora* (Surat Thani Province, Thailand), while (**1**) exhibited weak antibacterial activity against *Staphylococcus aureus* ATCC 25923 and methicillin-resistant *S. aureus* (MRSA) [47]. The intensity of antibacterial activity, such as weak, moderate, and strong, is mainly referred to in the published literature [48]. Two new naphthoquinone derivatives, 6-hydroxy-astropaquinone B (**2**) and astropaquinone D (**3**), as well as the three known quinones, 3-O-methyl-9-O-methylfusarubin (**4**), fusarubin (**5**), and javanicin (**6**), were isolated from the endophytic fungus *Fusarium napiforme,* which was obtained from the stem of *Rhizophora mucronata* collected from Makassar, South Sulawesi, Indonesia [49]. Of these, compounds **2**–**4** exhibited antibacterial activity against *Staphylococcus aureus* NBRC 13276 and *Pseudomonas aeruginosa* ATCC 15442, with MIC values of 20.8 µM for all tested strains, whereas compound **3** showed a weak activity against *S. aureus* (MIC = 41.3 µM). Compounds **5** and **6** exhibited antibacterial activity against *S. aureus,* with MIC values of 65.3 µM and 34.5 µM, respectively [49]. A known asperthecin (**7**) was isolated from the mangrove rhizosphere-associated fungus *Emericella* sp. strain SWR1718, which was obtained from the rhizosphere soil of *Avicennia marina*, collected along the Jeddah coastline in Saudi Arabia [50]. It exhibited weak cytotoxicity against the human lymphoma cell line HTB-176 (IC_50_ = 36.2 µM) and the human colorectal adenocarcinoma cell line HT-29 (IC_50_ = 82.8 µM), while showing lower activity against the human colon cancer cell line SW-620 (IC_50_ > 100 µM) [50].

Four known compounds, emodin (**8**), questin (**9**), physcion (**10**), questinol (**11**), and the novel acetylquestinol **14**, were isolated from the mangrove-derived endophytic fungus *Eurotium chevalieri* KUFA 0006 obtained from the inner twig of *Rhizophora mucronata* collected on the Eastern Seaboard of Thailand [51]. Further, the known anthraquinones **12** and **13** were isolated from the endophytic fungus *Stemphylium globuliferum* obtained from the mangrove plant *Avicennia marina* in Hurghada, Red Sea, Egypt [52]. In the same study, they reported the biological activities of identified compounds. For example, macrosporin (**12**) showed cytotoxic activity, with an IC_50_ value of 7.9 µM against the L5178Y mouse lymphoma cell line. And another study reported that the same compounds, **12** and **13**, were also isolated from *Phomopsis* sp. PSU-MA214, from the leaves of *Rhizophora apiculata* Griff. Ex T. Anderson from Songkhla province, Thailand [52]. Compound **12** exhibited antibacterial activity against *E. coli*, *Vibrio parahemolyticus*, and *Staphylococcus albus,* with MIC values ranging from 2.30 to 15 µM, whereas 1-hydroxy-3-methoxy-6-methylanthraquinone (**13**) demonstrated weak antibacterial activity, with a MIC of 186.3 µM against *S. aureus* [45,52]. Compound (**14**) exhibited antibacterial activity against *Staphylococcus aureus* ATCC 25923 (MIC = 93.5 µM) and *Enterococcus faecalis* ATCC 29212 (MIC = 187.0 µM) [51].

A novel phomopsanthraquinone (**15**) was isolated from *Phomopsis* sp. PSU-MA214, from the leaves of *Rhizophora apiculata* Griff. Ex T. Anderson from Songkhla province, Thailand [52]. This compound **15** demonstrated strong growth inhibition of MCF-7 cells, with an IC_50_ value of 81.3 µM [52]. Two new hydroanthraquinone derivatives, paradictyoarthrins A (**16**) and B (**17**), were isolated from the mangrove-derived fungus *Paradictyoarthrinium diffractum* BCC 8704, which was collected from mangrove wood in Laem Son National Park, Ranong Province, Thailand [53]. Compound (**16**), a chlorinated hydroanthraquinone, exhibited moderate cytotoxicity against KB, MCF-7, Vero, and NCI-H187 cell lines, with IC_50_ values of 61.4–82.7 µM. In contrast, compound **17**, a related hydroanthraquinone bearing a 4a,9a-epoxy functionality, displayed stronger cytotoxicity across all tested cancer cell lines, with IC_50_ values of 9.2–28.3 µM [53]. Moreover, known tetrahydroaltersolanols C (**18**), ampelanol, (**19**) and tetrahydroaltersolanol B (**20**) have been isolated from *Phomopsis* sp. PSU-MA214, from the leaves of *Rhizophora apiculata* Griff. Ex T. Anderson from Songkhla province, Thailand [52]. Compounds **18** and **19** showed selective antibacterial activity against *E. coli,* with MIC values of 9.8 and 7.3 µM, respectively, while **20** demonstrated activity against the fungi *Penicillium italicum* (MIC = 259.5 µM). The stereochemistry of compounds **18**, **19**, and **20** were determined by ROESY spectra and a modified Mosher’s method, establishing their absolute configurations as 2*S*,3*R*,4a*S*,9*S*,9a*S* for **18** [52]. Tetrahydroanthraquinone derivatives, altersolanol Q (**21**) and 10-methylaltersolanol Q (**22**) were isolated from the endophytic fungus *Stemphylium globuliferum* obtained from the mangrove plant *Avicennia marina* in Hurghada, Red Sea, Egypt [54]. The known quinones, dihydroaltersolanols B (**23**), C (**24**), altersolanols A (**25**), B (**26**), and N (**27**), were isolated from the endophytic fungus *Stemphylium globuliferum* obtained from the mangrove plant *Avicennia marina* in Hurghada, Red Sea, Egypt [54]. In those compounds, **25** was reported as the most potent, with cytotoxicity against the L5178Y mouse lymphoma cell line (IC_50_ = 5.2 µM), followed by **23** (IC_50_ = 8.4 µM), 37 (IC_50_ = 9.7 µM), 40 (IC_50_ = 12.6 µM), and **26** (IC_50_ = 15.3 µM) [54] (Figure 2).

Astronyquinone (**28**), isolated from the *Astrosphaeriella nypae* BCC 5335 obtained from the mangrove palm *Nypa fruticans*, exhibited weak cytotoxicity against Vero cells (IC_50_ = 57.6 µM) and demonstrated antituberculosis activity against *Mycobacterium tuberculosis* H37Ra, with a MIC value of 165.4 µM [55]. A known compound, TMC-264 (**29**), was isolated from the mangrove endophytic fungus *Penicillium chermesinum* strain HLit-ROR2 obtained from the leaves of *Xylocarpus granatum* collected at the Mangrove Forest Learning and Development Center 2, Samut Sakhon province, Thailand [56]. Dioxoauroglaucin (**30**), a novel prenylated benzaldehyde, a hydroxyanthrquinone derivative, was isolated from the mangrove-derived endophytic fungus *Aspergillus* sp. AV-2 obtained from the leaves of *Avicennia marina* in Hurghada, Red Sea, Egypt [57]. This compound demonstrated moderate antiproliferative activity against Caco-2 cells, with an IC_50_ value of 12.3 μM [57]. Altenusin (**31**), a known hydroxyanthraquinone derivative, was isolated from the mangrove-derived fungus *Paradictyoarthrinium diffractum* BCC 8704 obtained from mangrove wood in Laem Son National Park, Ranong Province, Thailand. This compound, **31**, exhibited weak cytotoxicity against the tested cancer cell lines, including NCI-H187, with IC_50_ values about 172.3 µM [53] (Figure 2). A new anthraquinone dimer, alterporriol X (**32**), along with known anthraquinone dimers (**33**–**37**), was isolated from the same endophytic fungus *Stemphylium globuliferum*. Alterporriols D, E, R, V, and W (**33**–**37**), which exhibited cytotoxic activities, with IC_50_ values ranging from 6.5 to 10.2 µM [54]. The structures of compounds **1**–**37** are presented in Figure 2. Accordingly, quinones isolated from marine fungi showed more cytotoxic activities. Therefore, there is great potential for developing them into novel anti-cancer agents.

When all bioactivities are considered, the most prominent bioactivity of quinones is antimicrobial activity. Therefore, these compounds can be developed into antibiotics, and they would be a promising solution for the prevalent antibiotic resistance.

#### 2.1.2. Naphthalenes

**Figure 3:**
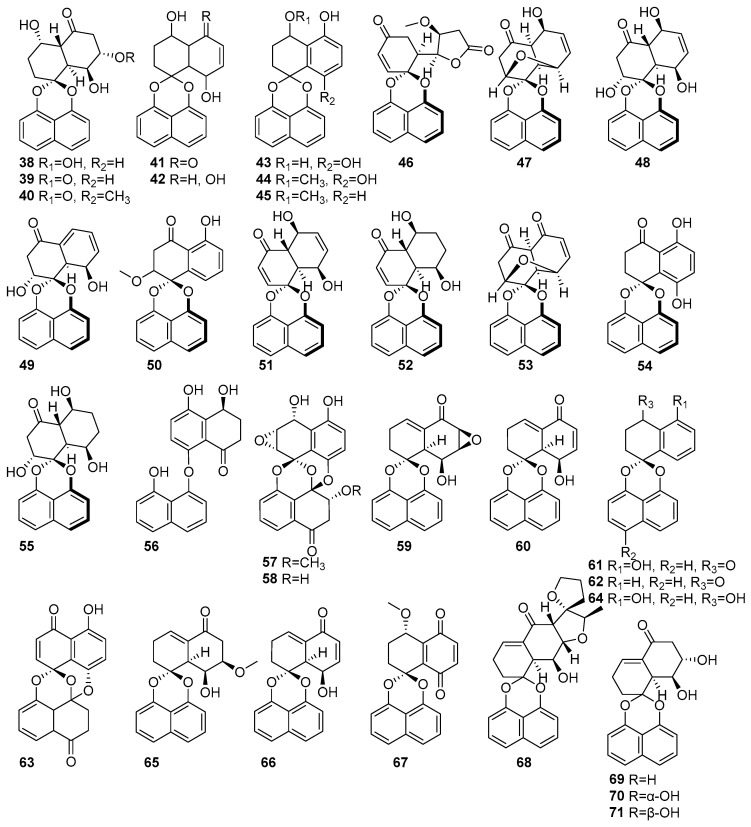
Naphthalenes in polyketide compounds isolated from mangrove fungi (**38**–**71**).

Figure 3 shows the naphthalene compounds in the polyketide groups isolated from mangrove fungi. Three new compounds, rhytidones A-C (**38**–**40**), and five known naphthalene compounds, MK3018 (**41**), palmarumycin CR1 (**42**), CJ-12,372 (**43**), 4-O-methyl-CJ-12,372 (**44**), 4-O-methyl-CJ-12,371 (**45)**, were isolated from the mangrove-derived endophytic fungus *Rhytidhysteron* sp. AS21B collected from the leaves of *Azima sarmentosa* in Samutsakhon Province, Thailand [43]. Compound **38** exhibited moderate cytotoxicity against MCF-7 and CaSki cell lines, with IC_50_ values of 14.47 and 21.95 μM, respectively. Compound **39**, featuring a ketone substitution at C-5, showed selective cytotoxicity towards CaSki cells (IC_50_ = 22.81 μM). Compound **40** displayed moderate cytotoxicity against both MCF-7 (IC_50_ = 17.30 μM) and CaSki (IC_50_ = 24.44 μM) cell lines. Compounds **41** and **42** exhibited moderate cytotoxicity against MCF-7 (IC_50_ = 14.47) and CaSki (25.59 μM) [43]. Moreover, **38**–**42** were also isolated from the endophytic fungus *Rhytidhysteron rufulum* AS21B and the leaves of *Azima sarmentosa* collected from the mangrove forest in Samutsakhon province, Thailand [58]. Compound **38** showed moderate anticancer activity against Ramos (IC_50_ = 23.1 µM) and weak activity against H1975 (IC_50_ = 50 µM) [58]. Further, compound **42** was also isolated from the mangrove-associated fungus *Rhytidhysteron* sp. AS21B obtained from *Azima sarmentosa* and collected in Samutsakhon Province, Thailand [59]. As a known spirobisnaphthalene lacking a carbonyl group at C-5, it was inactive in the nitric oxide inhibition assay yet retained moderate cytotoxicity (IC_50_ > 10 μM), highlighting the importance of the C-5 carbonyl moiety for cytotoxic activity [59]. Eleven spirodioxynaphthalenes derivatives, including five new palmarumycins P1–P5 (**46**–**50**), six known decaspirones A (**51**), C (**52**), palmarumycins CP_3_ (**53**), CP_17_ (**54**), M_2_ (**55**), and diaryl ether (**56**), were isolated from the mangrove-derived fungus BCC 25093 collected from unidentified mangrove wood at Hat Khanom, Mu Ko Thale Tai National Park, Surat Thani Province, Thailand. Compounds **46**–**50**, **53**, **55,** and **56** exhibited weak anti-malarial, antibacterial, and cytotoxic effects. Compounds (**51**) and C (**52**) showed moderate anti-malarial activity against *Plasmodium falciparum* K1, with IC_50_ values of 6.84 µM and 6.75 µM, respectively. Moreover, both **51** and **52** showed strong cytotoxicity against Vero cells, with IC_50_ values of 0.54 µM and 2.69 µM, respectively [60]. Known preussomerin C (**57**) and YMF 1029C (**58**) were isolated from mangrove-derived fungus *Paradictyoarthrinium diffractum* BCC 8704 obtained from mangrove wood collected at Laem Son National Park, Ranong Province, Thailand [53]. The spirocyclic metabolite **57** exhibited selective cytotoxicity against the NCI-H187 (small-cell lung cancer) cell line, with an IC_50_ value of 53.0 µM, and showed moderate cytotoxicity toward Vero cells (IC_50_ = 90.9 µM). Compound **58** showed strong cytotoxic effects, particularly against NCI-H187 cells, with an IC_50_ value of 13.1 µM. It also exhibited weak cytotoxic against KB and MCF-7 cell lines, with IC_50_ values of 34.0 and 99.4 µM, respectively [53] (Figure 3). This confirms that the anthracene derivatives derived from mangrove-associated fungi possess higher cytotoxic activity, and hence, development toward novel anticancer drugs would be a better option.

Several spirobisnaphthalene derivatives (**59**–**67**) were isolated from the endophytic fungus *Rhytidhysteron rufulum* AS21B habitats in the leaves of *Azima sarmentosa*, collected from the mangrove forest in Samutsakhon province, Thailand [58]. From this fungus, two new rhytidenones, G (**59**) and H (**60**), and the known deoxypreussomerin B (**61**) were identified. Compounds **59** and **61** exhibited moderate antitumor activity against human lymphoma Ramos cells (IC_50_ = 17.98 µM) and non-small-cell lung cancer H1975 cells (IC_50_ = 7.3 µM) [58]. In contrast, compound **60**, which contains an additional acetyl group at C-8, showed potent cytotoxicity, with IC_50_ values of 0.018 µM (Ramos) and 0.252 µM (H1975), showing the activity of standard drugs such as ibrutinib and afatinib [58]. New rhytidenone F (**66**), a deacetylated form of rhytidenone H, displayed strong antitumor activity, with IC_50_ values of 0.048 µM (Ramos) and 1.17 µM (H1975), making it the second-most-potent compound in the study. Other known derivatives, such as 1-oxo-1,4-dihydronaphthalene-4-spiro-2′-naphtho [4′,8′-dioxin] (**62**), preussomerin EG4 (**63**), CJ-12,371 (**64**), and novel rhytidenones E (**65**) and F (**66**), exhibited mild or weak activities [58]. Compound **66**, featuring an extended conjugated system, exhibited considerable cytotoxic activity, with an IC_50_ value of 4.90 µM, by inhibiting nitric oxide production in lipopolysaccharide-stimulated J774.A1 macrophage cells, as shown in Figure 3. The known compound palmarumycin C5, numbered as **67**, displayed weak activity against Ramos cells, with an IC_50_ value of 31.7 µM [58]. Rhytidenones A–D (**68**–**71**) were isolated from the mangrove-associated fungus *Rhytidhysteron* sp. AS21B obtained from *Azima sarmentosa* collected in Samutsakhon Province, Thailand [59]. The novel compound **68** exhibited strong anti-inflammatory activity, with an IC_50_ value of 0.31 µM in nitric oxide inhibition assays using lipopolysaccharide-stimulated J774.A1 macrophage cells. In contrast, compound **69**, an epimer at C-7 of **68**, showed moderate anti-inflammatory activity, with an IC_50_ value of 3.60 µM. Compound **70**, bearing hydroxyl groups at C-7 and C-8, demonstrated potent anti-inflammatory activity, with an IC_50_ value of 0.31 µM, and also exhibited notable cytotoxic effects. Compound **71**, a stereoisomer of **70** with altered 7-OH orientation, showed anti-inflammatory activity (IC_50_ = 3.60 μM) [59] (Figure 3). This information indicated the anti-inflammatory and cytotoxic activities of naphthalene compounds derived from mangrove fungi.

#### 2.1.3. Lactones and Macrolides

**Figure 4:**
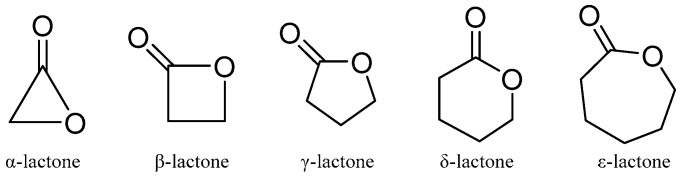
The basic chemical structures of lactones.

Lactones are outstanding exponents of secondary metabolites because of their remarkable biological activities and chemical architecture [61]. Natural lactones have a broad spectrum of biological activity, including antifeedant, antimicrobial, anti-inflammatory, and cytotoxic activities [61]. According to IUPAC, lactones are defined as cyclic esters of hydroxy carboxylic acids, containing a 1-oxacycloalkan-2-one structure, or analogs having unsaturation or heteroatoms replacing one or more carbon atoms of the ring. The smallest compounds of the class, α-, β-, γ-, δ-, and ω-lactones, have three-, four-, five-, six-, and seven-membered rings, respectively. The basic structure of lactones is shown in Figure 4.

**Figure 5:**
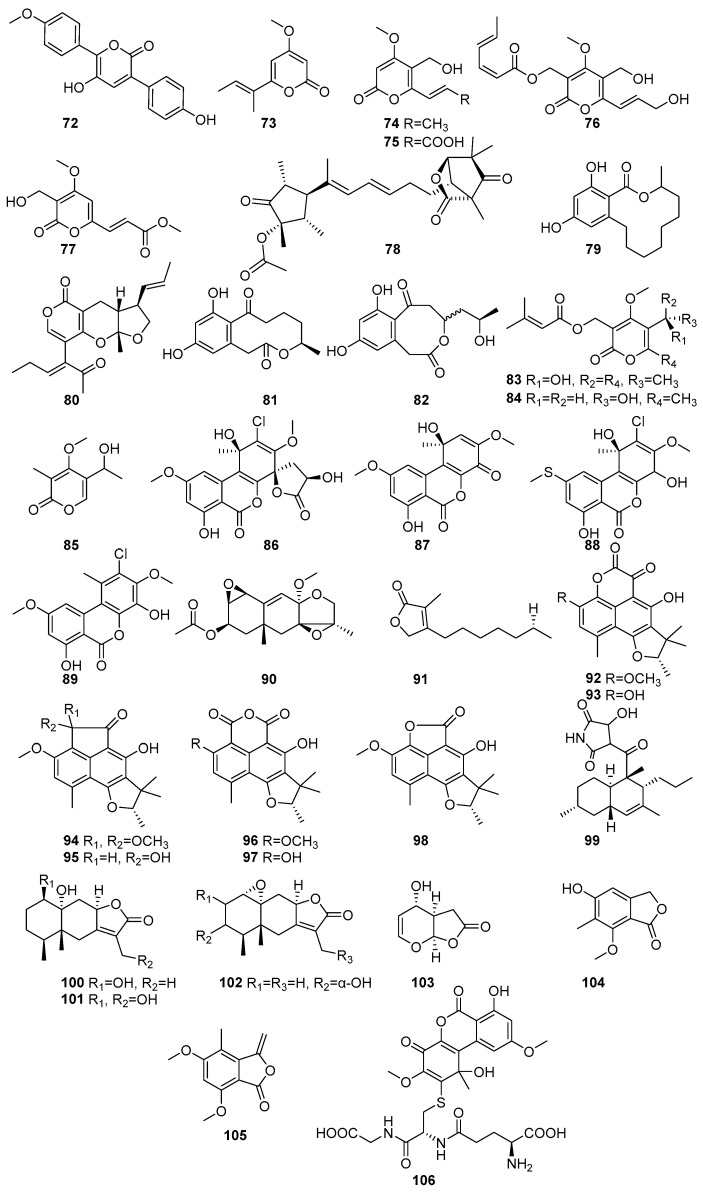
Lactones and macrolides in polyketide isolated from mangrove fungi (**72**–**106**).

**Figure 6:**
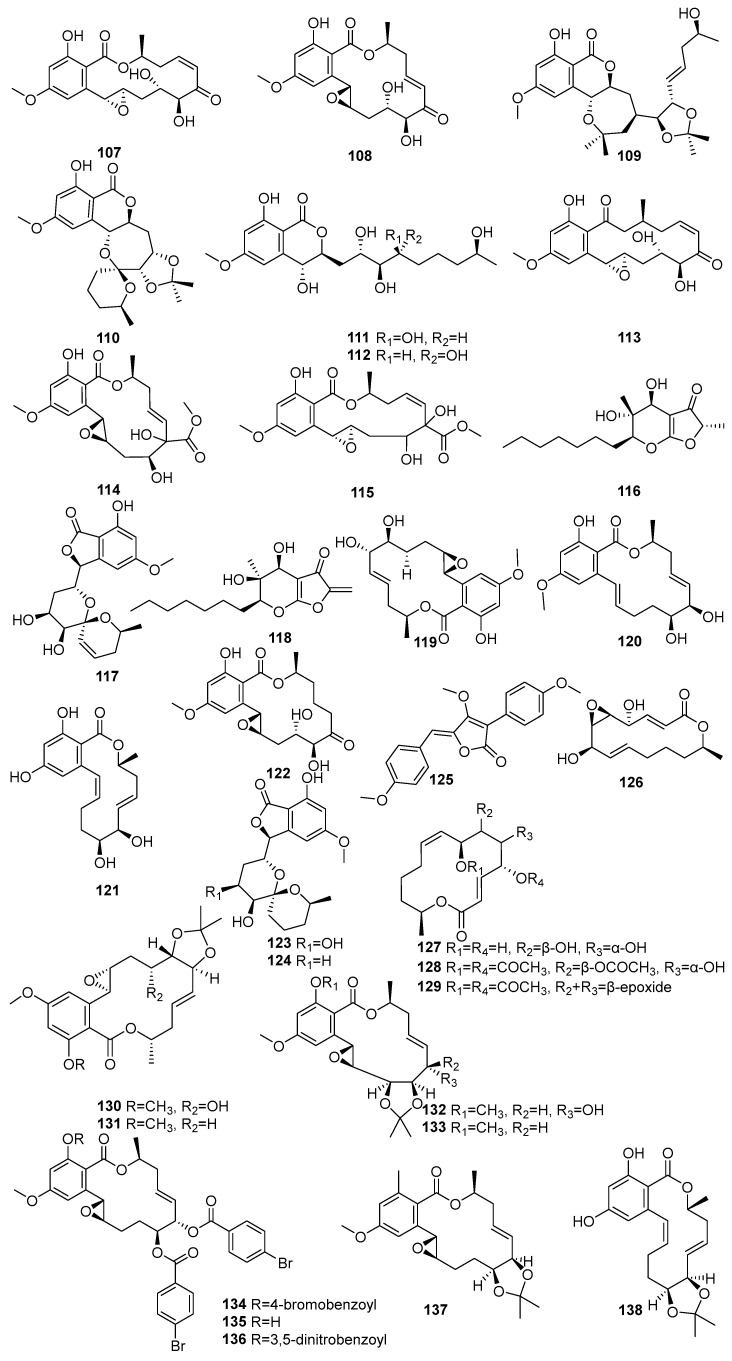
Some more lactone and macrolide in polyketide compounds isolated from mangrove fungi (**107**–**138**).

The lactones and macrolides isolated from different mangrove fungi are shown in Figure 5 (**72**–**106**) and Figure 6 (**107**–**138**). Known allantopyrone E (**72**), a lactone, was isolated from the endophytic fungus *Aspergillus versicolor* obtained from the *Avicennia marina* in Port Safaga, Red Sea Governorate, Egypt [62]. It exhibited cytotoxic activity against HeLa cells, with an IC_50_ value of 50.97 ± 1.7 μM [62]. Pestalopyrone (**73**) was isolated from the endophytic fungus *Nigrospora oryzae* obtained from the leaves of *Avicennia marina* collected from Kupang, East Nusa Tenggara, Indonesia [63]. Three known compounds, cladobotrin V (**74**), allantopyrone A (**75**), and islandic acid-II methyl ester (**76**), and a new α-pyrone derivative (**77**), were isolated from the endophytic fungus *Fusarium* sp. [64]. The *Fusarium* sp. IM-37 was obtained from the mangrove plant *Rhizophora mucronata* collected from Muara Angke, Jakarta, Indonesia [64]. Compounds **76** and **77** showed significant cytotoxic effects against HL60 cells, with IC_50_ values of 0.32 µM and 6.55 µM, respectively. In the Ca^2+^-signaling assay using the mutant yeast strain *Saccharomyces cerevisiae* YNS17, **75** exhibited a dose-dependent restoration of growth under Ca^2+^-induced stress conditions, suggesting its potential involvement in Ca^2+^-signaling pathways. Structural comparisons indicate that substitutions at the C-2 position influence the cytotoxic potency of **75** [64]. A novel Emericelactone E (**78**) was isolated from the mangrove rhizosphere-associated fungus *Emericella* sp. strain SWR1718 obtained from the rhizosphere soil of *Avicennia marina* collected from the Jeddah coastline, Saudi Arabia [50]. It exhibited moderate cytotoxic activity against the human lymphoma cell line HTB-176 (IC_50_ = 28.3 µM) and the human colon cancer cell line SW-620 (IC_50_ = 46.4 µM) [50]. The known compound 12,14-Dihydroxy-3-methyl-3,4,5,6,7,8,9,10-octahydro-1H benzo[c][1] oxacyclododecin-1-one (**79**) was isolated from the endolichenic fungus *Phanerochaete sordida* obtained from the lichen *Bactrospora myriadea* collected from the Negombo Lagoon, Sri Lanka [65]. It exhibited strong antioxidant activity in the ABTS assay (IC_50_ = 58.91 µM) and moderate anti-inflammatory activity (IC_50_ = 254.79 µM), stabilizing human red blood cell (HRBC) membranes. It also demonstrated moderate tyrosinase inhibition (IC_50_ = 17.13 × 10^2^ µM). Furthermore, **79** showed promising anticancer activity against the human oral cancer cell line CAL-27 (IC_50_ = 13.65 µM) [65]. Astronypyrone (**80**), xestodecalactone A (**81**), and ent-coryoctalactone B (**82**) were isolated from the mangrove-associated fungus *Astrosphaeriella nypae* BCC 5335 obtained from the mangrove palm *Nypa fruticans* collected from Samut Prakan Province, Thailand [66]. Compound **80** exhibited strong antioxidant activity (IC_50_ = 59.5 µM) and moderate antibacterial activity against *Bacillus subtilis* (MIC = 80.1 µM). In contrast, **82** showed notable antioxidant activity (IC_50_ = 108.5 µM) and moderate cytotoxicity against Vero cells (IC_50_ = 56.4 µM) [66]. Pestalotiopyrones A–C (**83**–**85**) were isolated from the mangrove-derived fungus *Pestalotiopsis* sp. PSU-MA92 obtained from the twigs of *Rhizophora apiculate* and collected from Trang Province, Thailand [67]. Novel compound **83** exhibited moderate antibacterial activity against *Bacillus subtilis* (MIC = 154.2 µM), while **84** showed weak activity against *Staphylococcus aureus* (MIC = 197.4 µM) [67]. Penicilliumolides A–E (**86**–**90**) were isolated from the mangrove endophytic fungus *Penicillium chermesinum* strain HLit-ROR2 obtained from the leaves of *Xylocarpus granatum* and collected in Thailand. Penicilliumolides B (**87**) and C (**88**) exhibited weak cytotoxicity against MOLT-3 and T47D cell lines [58]. A novel butenolide derivative, pestalolide (**91**), was isolated from the *Pestalotiopsis* sp. PSU-MA69 obtained from the mangrove plant *Rhizophora apiculata* collected in Sutun province, Thailand [68]. In antifungal screening, **91** showed weak inhibitory activity against both *Candida albicans* NCPF3153 and *Cryptococcus neoformans* ATCC90112, with minimum inhibitory concentration (MIC) values of 652.3 µM for each strain [68]. The compounds (−)-Bipolaride A (**92**), (−)-scleroderolide (**93**), (−)-bipolarides B (**94**), C (**95**), (−)-sclerodin methyl ether (**96**), (−)-sclerodin (**97**), (−)-bipolaride E (**98**), and oxasetin (**99**) were isolated from the marine-derived fungus *Lophiostoma bipolare* BCC 25910 obtained from mangrove wood collected at Haad Wanakorn National Park, Thailand [69]. Compounds **92**, **95**, and **98** exhibited moderate antimicrobial activity against *Bacillus cereus*, each with MIC values of 36.5 µM, 31.2 µM, and 39.8 µM, respectively, while **94** showed weaker activity (MIC = 62.4 µM). Compound **93** demonstrated antimicrobial activity against *Staphylococcus aureus* SG511 with a MIC of 24 μM, while compound **97** inhibited human leukocyte elastases, with an IC_50_ of 10.9 μM. All **92**–**98** compounds showed weak cytotoxicity against human cancer cell line MCF-7 (146.1 µM, 56.3 µM, 120.6 µM, 124.8 µM, 108.3 µM, 152.4 µM, 146.1 µM, respectively) [69]. Polyketide lactones, Mairetolide F (**100**), 13-hydroxymairetolide F (**101**), and 3α-hydroxymairetolide A (**102**), with weak results in antibacterial, antimalarial, and cytotoxic assays, were isolated from the mangrove-derived fungus *Xylariaceae* sp. BCC 60405 obtained from mangrove wood collected in Ko Hua Ta Chio, Trat Province, Thailand [70]. Oxysporone (**103**) has been isolated from the mangrove-derived endophytic fungi *Pestalotia* sp. obtained from the leaves of *Heritiera fomes* collected in the Sundarbans mangrove forest, Bangladesh [71]. It demonstrated notable anti-MRSA activity, with MIC values ranging from 32 to 128 µM, showing particular effectiveness against EMRSA-15 and SA-1199B, both with an MIC of 32 µM, indicating its potential as an anti-MRSA therapeutic [71]. A novel compound named nidulol (**104**) was isolated from the mangrove rhizosphere-associated fungus *Emericella* sp. strain SWR1718 obtained from the rhizosphere soil of *Avicennia marina* collected from the Jeddah coastline, Saudi Arabia. It exhibited moderate cytotoxicity against the human cell lines HTB-176, SW-620, and HT-29, with the IC_50_ values of 18.6 µM, 15.7 µM, and 36.9 µM, respectively [50]. A new phthalide derivative, acremonide (**105**), was isolated from the mangrove-derived endophytic fungus *Acremonium* sp. PSU-MA70 obtained from a branch of *Rhizophora apiculata* collected in Satun Province, Thailand [72]. A known compound, penicilliumolides G (**106**), was isolated from the mangrove endophytic fungus *Penicillium chermesinum* strain HLit-ROR2 obtained from the leaves of *Xylocarpus granatum* and collected at Mangrove Forest Learning and Development Center 2, Samut Sakhon province, Thailand [56]. Compound **106** as a well-known mycotoxin and demonstrated strong cytotoxicity against HL-60 (IC_50_ = 0.06 µM), HuCCA-1 (IC_50_ = 2.19 µM), HeLa (IC_50_ = 1.87 µM), T47D (IC_50_ = 0.81 µM), and MDA-MB-231 (IC_50_ = 1.36 µM), with a selectivity index of 61 for HL-60, highlighting its selective anticancer potential against leukemia cells [56] (Figure 5).

Hypothemycin (**107**) and aigialomycin A (**108**) were isolated from *Aigialus parvus* BCC 5311, an endophytic fungus derived from mangrove wood collected in Thailand [73]. Compound **107** exhibited moderate antimalarial activity against *Plasmodium falciparum* K1 (IC_50_ = 5.8 µM) and showed cytotoxicity toward KB, BC-1, and Vero cells, with IC_50_ values of 44.9 µM, 16.4 µM, and 16.6 µM, respectively. Compound **108**, an isomer of **107** with a trans C-7′–C-8′ double bond, was weak in the antimalarial assay (IC_50_ > 52.9 µM), but exhibited selective cytotoxicity, particularly against Vero cells (IC_50_ = 11.4 µM) [73]. Another study showed known **107**, and five novel compounds **108**–**112**, the acetonide derivative of aigialomycin F (**109**), aigialomycin G (**110**), 7′,8′-dihydroaigialomycin F (**111**), and **112** resorcylic acid lactones, which were identified from the mangrove-derived endophytic fungus *Aigialus parvus* BCC 5311 isolated from a mangrove habitat in Thailand [74]. Compound **107** was identified as a prototypical resorcylic acid lactone with potent protein kinase inhibitory activity. It demonstrated moderate antimalarial activity against *Plasmodium falciparum* K1 (IC_50_ = 7.4 µM), and selective cytotoxicity against NCI-H187 (IC_50_ = 5.3 µM) and Vero cells (IC_50_ = 5.6 µM) [74].

A new aigialomycin B (**113**) was isolated from the mangrove-derived fungus *Aigialus parvus* BCC 5311, which was collected from mangrove wood in Thailand [73]. Two new compounds, a rearranged resorcylic acid lactone derivative of aigialomycin A (**108**) and its stereoisomer (**114**,**115**), were isolated from the mangrove-derived endophytic fungus *Aigialus parvus* BCC 5311 obtained from a mangrove habitat in Thailand [74]. The known aigialone (**116**) was unlike the resorcylic acid lactones and spiroacetal macrolides isolated from *Aigialus parvus* BCC 5311 [74]. Another study reported that **116**, aigialospirol (**117**), and benesudon (**118**) were isolated from the mangrove-derived fungus *Aigialus parvus* BCC 5311, which was obtained from mangrove wood in Thailand [75]. The new aigialomycins C–E (**119**–**121**) and known dihydrohypothemycin (**122**) were isolated from *Aigialus parvus* BCC 5311, an endophytic fungus derived from mangrove wood collected in Thailand [73]. Compound **120**, containing a trans-olefin at C-1′–C-2′ and a free phenolic OH at C-4, demonstrated moderate antimalarial activity (IC_50_ = 19.7 µM) and potent cytotoxicity against KB and Vero cells, with IC_50_ values 9.0 and 5.4 µM, respectively [73].

7′,8′-Dihydroaigialospirol (**123**) and 4′-deoxy-7′,8′-dihssydroaigialospirol (**124**) were isolated from the mangrove-derived endophytic fungus *Aigialus parvus* BCC 5311 obtained from a mangrove habitat in Thailand. Both failed to demonstrate any antimalarial or cytotoxic effects [74]. A new pulvinone derivative, dimethoxy-O-methylpulvinone (**125**), was isolated from the *Astrosphaeriella nypae* BCC 5335. Compound **125** exhibited potent antimalarial activity against *Plasmodium falciparum* K1 (IC_50_ = 17.7 µM) and moderate cytotoxicity against Vero cells (IC_50_ = 58.6 µM) [55]. Two novel pestalotioprolides, B (**126**) and A (**127**), known **125**, a triacetate derivative of pestalotioprolide A (**128**), and a diacetate derivative of seiricuprolide (**129**) were isolated from the fermentation extracts of *Pestalotiopsis* spp. PSU-MA119 and PSU-MA92 obtained from *Nypa fruticans* and *Rhizophora apiculata* collected in Thailand [67]. Nine acetonide derivatives (**130**–**138**) were isolated from *Aigialus parvus* BCC. 5311, an endophytic fungus derived from mangrove wood collected in Thailand. Among them, only **130**, featuring a modified C-7 side chain, showed moderate cytotoxicity towards KB and BC-1 cells (IC_50_ = 19.7 µM and 53.7 µM, respectively) [73] (Figure 6). Accordingly, a large number of lactone macrolides have been identified from mangrove fungi grown on the Indian Ocean coast, and their dominant activity is cytotoxic. In addition, some compounds with anti-malarial activity have been identified in these compounds, showing the potential for development into a novel treatment for malaria.

#### 2.1.4. Phenolic Compounds

**Figure 7:**
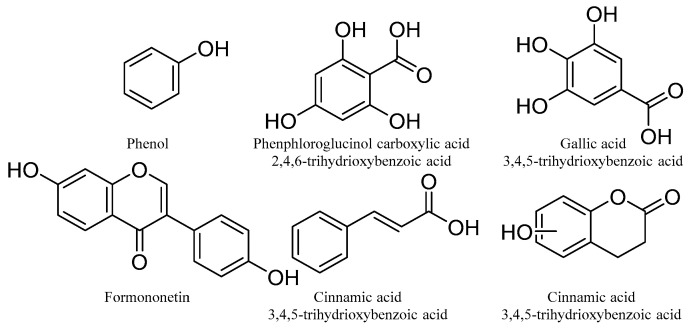
The basic chemical structures of phenolic compounds.

Phenolic compounds are defined as natural secondary metabolites characterized by hydroxylated aromatic rings, which may include one or more hydroxy groups directly linked to phenyl or substituted phenyl groups [76]. Phenolic compounds are the most abundant secondary metabolites in plants and are responsible for a range of bioactivities, including being strong antioxidants [77]. Phenolic compounds are known to be associated with a reduction in the risk of non-communicable diseases through minimizing excessive oxidative stress generated in biological cells [78,79]. Phenolic compounds show a wide range of structural diversity and, hence, diversity of their pharmacological properties. The basic structures of phenolic compounds are shown below (Figure 7), which include phenols, polyphenols, phenolic acids, flavonoids, etc.

**Figure 8:**
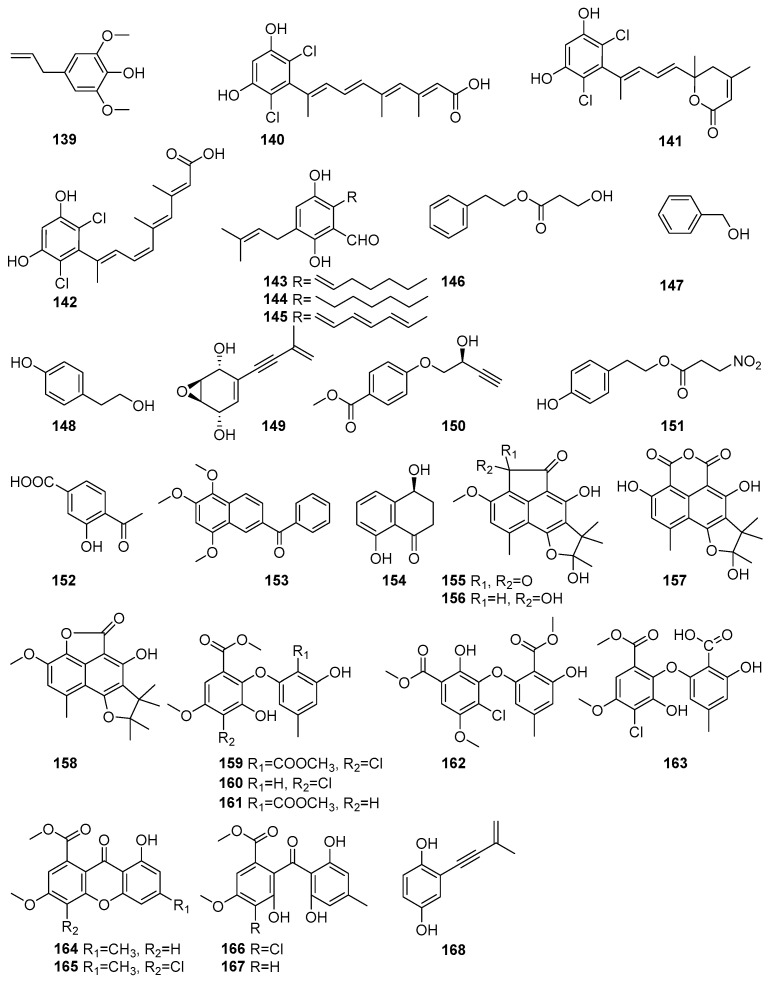
Phenolic compounds in polyketide isolated from mangrove fungi (**139**–**168**).

Biologically important phenolic compounds have been isolated from mangrove-associated fungi, and their biological activities have been reported. Figure 8 shows phenolic compounds in polyketides characterized from mangrove fungi (**139**–**168**). The compound 2,6-Dimethoxyphenol (**139**) was isolated from the *actinomycete* strain SMS_SU21 collected from the Sundarbans mangrove forest, Bangladesh [80]. Compound **139** exhibited strong antimicrobial activity against *Candida albicans*, with a MIC value of 324µM. This strain also showed moderate antioxidant properties, which had never been previously reported for *Streptomyces coelicolor* [80]. Three novel dichlorinated metabolites, cosmochlorins A–C (**140**–**142**), were isolated from the fermentation extract of the endophytic fungus *Cosmospora vilior* IM2-155 obtained from the mangrove plant *Sonneratia alba* collected in Pagandaran, West Java, Indonesia [81]. Compound **140** exhibited antimicrobial activity against *Trichoderma harzianum* (MIC = 42.3 µM)*, Aspergillus clavatus* (MIC = 169.3 µM), and *Candida albicans* (MIC = 338.7 µM). It also inhibited GSK-3β, with an IC_50_ value of 62.5 µM. Compound **141** showed weak antimicrobial activity (MIC > 338.7 µM) but inhibited GSK-3β, with an IC_50_ of 60.6 µM, and enhanced osteoclast formation by over 1.5-fold in RAW264.7 cells, indicating potential in bone metabolism regulation. Compound **142**, a structural isomer of **139**, showed moderate activity against *T. harzianum* (MIC = 48.0 µM) [81]. Tetrahydroauroglaucin (**143**), flavoglaucin (**144**), and auroglaucin (**145**) were isolated from the fermentation extract of the acid-tolerant mangrove sediment-derived fungus *Penicillium oxalicum* OUCMDZ-5207 of PakMeng Beach, Thailand [82]. Compound **143**, a prenylated benzaldehyde derivative, exhibited significant cytotoxic activity against human lung adenocarcinoma (A549) and breast adenocarcinoma (MCF-7) cell lines, with an IC_50_ value of 5.67 µM against A549 cells [82]. Compound **144** demonstrated moderate cytotoxic effects, with inhibition rates of 32% (A549) and 27% (MCF-7) at 10 µM concentration. Compound **145** showed the strongest cytotoxic activity, with 79% inhibition of A549 cells and 48% inhibition of MCF-7 cells at 10 µM, and an IC_50_ value of 5.67 µM, comparable to that of the positive control adriamycin (IC_50_ = 0.61µM) [82]. A known phenethyl alcohol, hydracrylate (**146**), was isolated from the mangrove-derived fungus *Phomopsis* sp. PSU-MA214 obtained from the leaves of *Rhizophora apiculate* and collected in Songkhla Province, Thailand [52]. Benzene ethanol (**147**) and 4-hydroxy benzeneethanol (**148**) were identified from the endophytic fungus *Xylaria feejeensis* strain AML-02 obtained from *Avicennia marina* leaves collected at Wat Asokaram, Samut Prakan, Thailand [83]. Known diphenyl ether derivative compounds, namely asperpentyn (**149**) and (S)-penipratynolene (**150**), were isolated from the *Pestalotiopsis* sp. PSU-MA69, an endophytic fungus associated with the mangrove plant *Rhizophora apiculata* collected in Thailand [68]. Compound **149** features a polyoxygenated framework, while **150** is a polyene-type metabolite bearing a conjugated alkyne chain and a hydroxylated chiral center [68]. A known phomonitroester, (**151**), an aromatic ester, was isolated from the mangrove-associated fungus *Phomopsis* sp. PSU-MA214 obtained from a leaf of *Rhizophora apiculata* collected in Songkhla Province, Thailand [52]. Compound **151** exhibited selective cytotoxicity against the KB oral carcinoma cell line, with an IC_50_ value of 179.8 µM, but showed no cytotoxic effect on MCF-7 and Vero cell lines. Additionally, phomonitroester **151** exhibited moderate antibacterial activity against *Staphylococcus aureus* ATCC 25923, with an MIC value of 418.1 µM [52]. A novel compound, 3-hydroxy-4-(1-oxo-ethane) benzoic acid **152**, was isolated from the endophytic fungus *Aspergillus versicolor* obtained from the mangrove plant *Avicennia marina* in the Red Sea, Egypt. Compound **152** displayed cytotoxic activity against HeLa cells, with an IC_50_ value of 53.5 μM [84]. A new naphtho-phenyl ketone derivative, nigronapthaphenyl (**153**), was isolated from the endophytic fungus *Nigrospora sphaerica* obtained from the mangrove plant *Bruguiera gymnorrhyza* collected from the Attaragoda Wetland, Galle, Sri Lanka [85]. It exhibited broad-spectrum antibacterial activity against *Bacillus subtilis* TISTR 088, *Bacillus cereus* TISTR 688, *Staphylococcus aureus* ATCC 43300, *Escherichia coli* UBC 8161, and methicillin-resistant *Staphylococcus aureus* (MRSA) ATCC 33591, with MIC values of 5.3–10.7 µM. Additionally, **153** demonstrated moderate cytotoxic activity against the human colon cancer cell line HCT 116 (IC_50_ = 9.62 ± 0.5 µM), significant anti-inflammatory activity by inhibiting IL-6 release (IC_50_ = 6.2 ± 0.5 µM), and α-glucosidase inhibitory activity (IC_50_ = 6.9 ± 0.5 µM) [85]. A known compound, named isosclerone (**154**), was isolated from the mangrove-derived fungus *Xylaria cubensis* PSU-MA34 obtained from a branch of *Bruguiera parviflora* collected in Surat Thani Province, Thailand [48]. Compound **154**, with mild antimicrobial activity, was also isolated from the mangrove-derived endophytic fungus *Daldinia eschscholtzii* PSU-STD57 obtained from a leaf of *Bruguiera gymnorrhiza* collected in Suratthani Province, Thailand [86] (Figure 8).

Further, more phenolic compounds, four new bipolarol derivatives, bipolarols A–D (**155**–**158**), were isolated from the fungus *Lophiostoma bipolare* BCC25910 collected from mangrove wood at Haad Wanakorn National Park, Thailand [69]. These bipolarol derivatives differ structurally in their substitution patterns at C-20, which influenced their bioactivity. Compound **157** exhibited weak antimicrobial activity against *Bacillus cereus* (MIC = 62.4 µM). The cytotoxicity assays showed that **156** displayed moderate activity against KB (IC_50_ = 52.5 μM), MCF-7 (IC_50_ = 65.3 μM), and NCI-H187 (IC_50_ = 48.3 μM), while **158** and **155** were weakly cytotoxic, with IC_50_ values > 100 μM for all cell lines tested [69]. Four new diphenyl ethers, pestalotethers A–D (**159**–**162**) and the known compounds, pestheic acid (**163**), isosulochrin dehydrate (**164**), chloroisosulochrin dehydrate (**165**), chloroisosulochrin (**166**), and isosulochrin (**167**), along with siccayne (**168**), were isolated from *Pestalotiopsis* sp. PSU-MA69, an endophytic fungus derived from the mangrove plant *Rhizophora apiculata* collected in Thailand [68]. The novel chlorinated diphenyl ether derivatives, **159** and **160**, exhibited mild antifungal activity against *Cryptococcus neoformans,* with MIC values of 595.0 µM. The known compounds **163** and **165** demonstrated mild antifungal activity against *C. neoformans*, whereas compounds **164** and **166**–**168** were found to be inactive against both *C. albicans* and *C. neoformans* [68] (Figure 8). These findings showed the potent antimicrobial activities and cytotoxicity of the phenolic compounds present in mangrove fungi from the Indian Ocean coast.

#### 2.1.5. Coumarins and Chromone Derivatives

**Figure 9:**
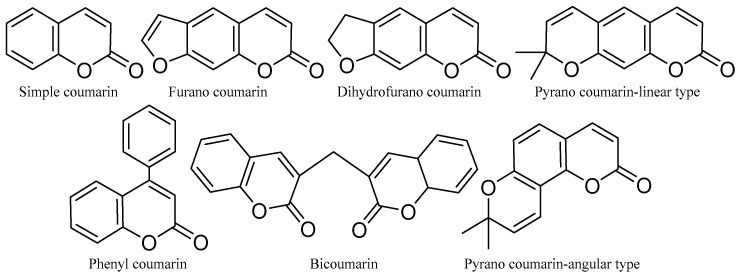
The basic chemical structures of coumarins.

Coumarins are a family of benzopyrones that represent an important family of oxygen-containing heterocycles and are widely distributed in nature [87]. These molecules are very special due to their conjugated double-ring system [88]. They are simple and can be structurally modified into important compounds. Coumarins have a wide range of bioactivities and pharmaceutical properties, including anti-cancer, anti-microbial, etc. [89], and are used in the pharmaceutical industry for the synthesis of a large number of synthetic pharmaceuticals. Coumarins are also found in industry as cosmetics and perfume ingredients, as food additives, and especially in the pharmaceutical industry in the synthesis of many synthetic pharmaceutical products [90]. The basic structure of coumarins is shown below (Figure 9).

**Figure 10:**
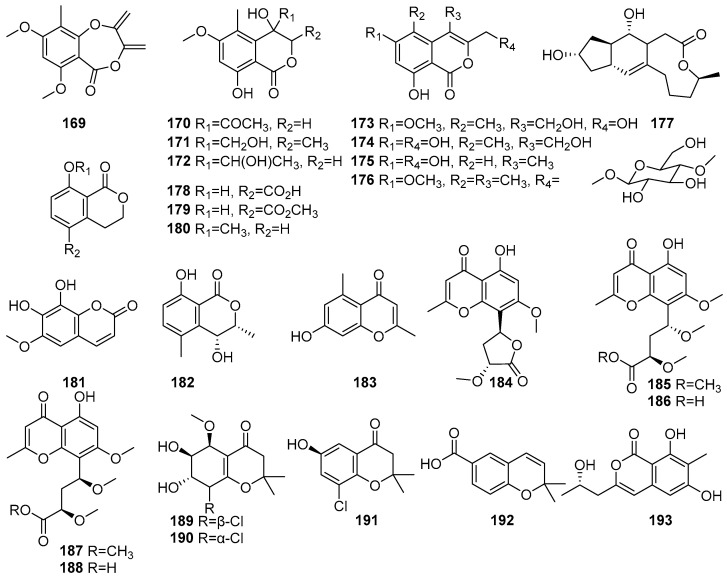
Coumarins and chromone derivatives in polyketide isolated from mangrove fungi (**169**–**193**).

Mangrove-derived fungi have been reported as sources of coumarin derivatives with biological activities. Figure 10 shows coumarin derivatives of polyketides isolated from mangrove fungi (**169**–**193**). The *Acremonium* sp. PSU-MA70, isolated from a branch of *Rhizophora apiculata* collected in Satun Province, Thailand, is reported as a source of eight new isocoumarin derivatives, acremonones A–H (**169**–**176**) [72]. The known (+)-Brefeldin A **177** was isolated from *Acremonium* sp. PSU-MA70, obtained from a branch of mangrove *Rhizophora apiculata* collected in Satun Province, Thailand. Compound **177** demonstrated moderate antifungal activity against *C. albicans,* with an MIC value of 114.2 µM, while its activity against *C. neoformans* was weak [72]. Compounds (R)-(−)-5-Fraxetin (**178**), (R)-(−)-5-methoxycarbonylmellein (**179**), and (R)-(−)-mellein methyl ether (**180**) have been isolated from the fermentation extract of the mangrove-derived fungus *Xylaria cubensis* PSU-MA34 obtained from a branch of *Bruguiera parviflora* collected in Surat Thani Province, Thailand [48]. Fraxetin (**181**), a known hydroxycoumarin derivative, was isolated from the *Aspergillus fumigatus*, a mangrove-derived endophytic fungus from *Ceriops decandra*, collected from the Sundarbans mangrove forest, Bangladesh. It exhibited antibacterial activity against *Staphylococcus aureus,* with an MIC value of 30 × 10^2^ µM [91]. A known compound named (3R,4R)-4-Hydroxy-5-methylmellein (**182**) was isolated from *Xylariaceae* sp. BCC 60405 obtained from mangrove wood collected at Ko Hua Ta Chio, Trat Province, Thailand [70] (Figure 10). Accordingly, coumarin compounds isolated from fungi are reported to show moderate antimicrobial activity.

The chromones constitute an important class of oxygen-containing heterocyclic compounds, many of which are widely distributed in plants and possess useful medicinal properties [92]. Chromone skeleton-containing molecules have been found to possess various biological activities, including antimicrobial, antiallergenic, antiviral, antihypertensive, anti-inflammatory, antitumor, etc. [93]. Compounds **183**–**193** are identified as chromone derivatives in polyketides isolated from mangrove fungi. A known chromone derivative, Altechromone A (**183**), was isolated from the endophytic fungus *Stemphylium globuliferum* obtained from the mangrove plant *Avicennia marina* in Hurghada, Red Sea, Egypt, and exhibited moderate cytotoxicity, with an IC_50_ value of 14.5 µM against the L5178Y mouse lymphoma cell line [48]. Five new chromones, named rhytidchromones A–E (**184**–**188**), were isolated from the mangrove-derived endophytic fungus *Rhytidhysteron rufulum* obtained from the mangrove plant *Bruguiera gymnorrhiza* collected from Pak Nam Pran, Prachuab Kiri Khan, Thailand [66]. Compound **184** was identified as a highly oxygenated chromone derivative and exhibited cytotoxic activity against the Kato-3 and MCF-7 breast cancer cell lines, with IC_50_ values of 23.3 µM and 19.3 µM, respectively. Compounds **185** and **186** showed cytotoxicity against the Kato-3 cell line, with IC_50_ values of 21.4 µM and 16.8 µM, respectively [66]. Compound **188** showed cytotoxic activity against Kato-3 (IC_50_ = 16.0 µM) and MCF-7 (IC_50_ = 17.7 µM) [66]. Three new chlorinated chromone derivatives, pestalochromones A–C (**189**–**191**), and a known compound, 2,2-dimethyl-2H-1-chromene-6-carboxylic acid (**192**), were isolated from the *Pestalotiopsis* sp. PSU-MA69, an endophytic fungus associated with the mangrove plant *Rhizophora apiculata*, collected in Thailand [68]. A novel compound, **193**, identified as 5, 6,8-dihydroxy-3-(2S-hydroxypropyl)-7-methyl-1H-isochromen-1-one, was isolated from the mangrove-derived endophytic fungus *Eurotium chevalieri* KUFA 0006 obtained from *Rhizophora mucronata* collected in Thailand. Compound **193** exhibited a moderate inhibition of biofilm formation by *Escherichia coli* ATCC 25922, with a 50.6% reduction at 255.7 µM [51] (Figure 10). These chromone compounds show moderate-to-high cytotoxicity, showing their potential for developing cytotoxic compounds in a therapeutic regime of phenolic compounds present in mangrove fungi from the Indian Ocean coast.

#### 2.1.6. Xanthone and Benzofurans

**Figure 11:**
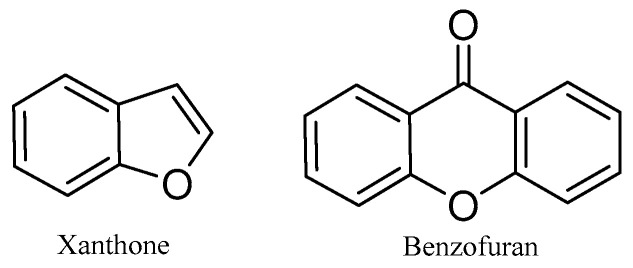
The basic chemical structures of xanthone and benzofuran.

Both xanthones and benzofurans are heterocyclic compounds under the polyketides category and are reported to possess biological activities [1]. They are synthesized in certain plants, fungi, and bacteria. The basic structures of xanthones and benzofurans are shown in Figure 11.

**Figure 12:**
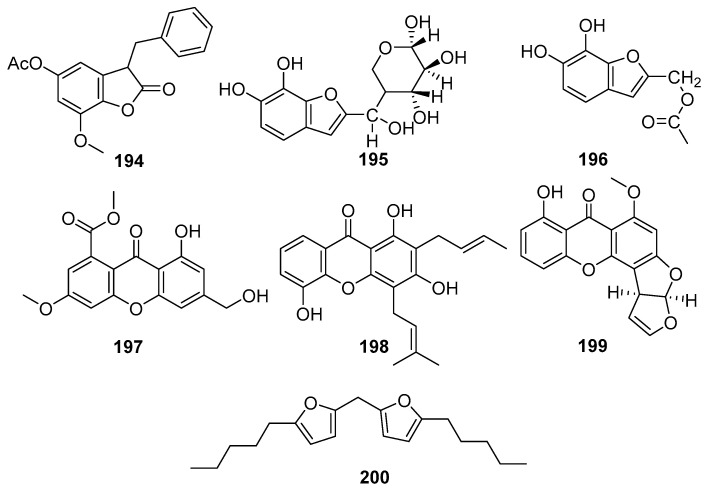
Xanthones and benzofuran compounds in polyketide isolated from mangrove fungi (**194**–**200**).

Structurally diverse and biologically active xanthones and benzofurans are reported from mangrove-associated fungi. Figure 12 shows xanthone and benzofuran compounds derived from mangrove fungi (**194**–**200**). A new benzofuranone derivative, sonneratinone (**194**), was isolated from the *Aspergillus niger* fungus collected from the Sundarbans mangrove forest, Bangladesh [94]. It exhibited significant antimicrobial activity, with MIC values of 31.1 µM against *Micrococcus luteus* and *Staphylococcus aureus*, a MIC value of 80 µM against *Pseudomonas aeruginosa*, and a MIC value of 160.1 µM against *Candida albicans* [94]. Two novel benzofuranoids, deuteromycols A (**195**) and B (**196**), were isolated from the marine-derived fungal strain *Deuteromycete* sp. MF003 obtained from mangrove driftwood collected on the shore of the Red Sea, El Gouna, Egypt [95]. A novel xanthone derivative, pestaloxanthone (**197**), was isolated from the *Pestalotiopsis* sp. PSU-MA69, an endophytic fungus associated with the mangrove plant *Rhizophora apiculata*, collected in Thailand [96]. Accordingly, compound **197** was reported to exhibit weak antifungal activity against both *Candida albicans* NCPF3153 and *Cryptococcus neoformans* ATCC90112, with MIC values of 387.6 µM for each strain [68]. A series of compounds containing oxygen heterocycles, namely furan derivatives, were identified as fungal metabolites [96].

Usually, furan derivatives are toxic or become carcinogenic when they are oxidized [97]. The coumarin 8-Deoxytrichothecin (**198**) showed moderate antifungal activity against *C. albicans* (MIC = 48.5 µM) but was weakly active against *C. neoformans* (MIC ≥ 387.6 µM) [72]. A new bioactive polyketide derivative, Sterigmatocystin (**199**), was isolated from the endophytic fungus *Nigrospora oryzae* obtained from the leaves of *Avicennia marina* collected from Kupang, East Nusa Tenggara, Indonesia [43]. Compound **199** exhibited moderate cytotoxic activity against the murine lymphoma L5178Y cell line, inhibiting cell proliferation by 64% at a concentration of 30.8 µM, and the structural analogs such as 5-methoxydihydrosterigmatocystin, have been reported to possess potent antibacterial activity and antiparasitic effects against *Trypanosoma cruzi* [98], suggesting that **221** could be further developed as an antibacterial and antiviral agent [43] (Figure 12). A novel difuranylmethane derivative, flavodonfuran (**200**), was isolated from the mangrove-derived endophytic fungus *Flavodon flavus* PSU-MA201 obtained from the leaf of *Rhizophora apiculata* collected from Thailand [99].

#### 2.1.7. Fatty Acid Derivatives and Other Polyketides

**Figure 13:**
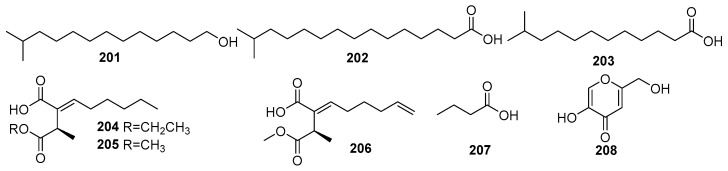
Derivatives of fatty acid in polyketide isolated from mangrove fungi (**201**–**208**).

Some fatty acid derivatives are secondary metabolites synthesized in fungi and share a common biosynthetic pathway as polyketides [1]. Therefore, these are discussed under the polyketides category of fungal metabolites. For fungi, the metabolism of fatty acid is associated with important processes of the fungal life cycle, such as spore formation. The mangrove rhizosphere soil-derived fungi are one of the major groups of biological organisms that provide a significant number of unsaturated fatty acids with pharmacological properties [100]. Figure 13 shows the derivatives of fatty acids isolated from mangrove fungi (**201**–**208**). The known compounds, 12-methyltetradecanoic acid (**201**), palmitic acid (**202**), and tridecanoic acid (**203**), were isolated from the marine actinobacterium *Streptomyces albus* MAB56 obtained from mangrove sediments in the Andaman Islands, India [101]. Compound **201** exhibited strong antibacterial activity against *Staphylococcus aureus* (MIC = 12.9 µM) and moderate activity against *Escherichia coli* (MIC = 51.6 µM), whereas **202** showed antibacterial effects against *S. aureus* (MIC = 24.4 µM) and *E. coli* (MIC = 97.5 µM) and demonstrated anti-HIV activity, with an IC_50_ value of <3.9 µM. Compound **203** exhibited antibacterial activity against *S. aureus* (MIC = 58.3 µM) and *E. coli* (MIC = 116.7 µM) [101]. Two novel succinic acid derivatives, namely xylacinic acids A (**204**) and B (**206**), and a known 2-hexylidene-3-methylsuccinic acid 4-methyl ester (**205**), were isolated from the mangrove-derived fungus *Xylaria cubensis* PSU-MA34 obtained from a branch of the mangrove plant *Bruguiera parviflora* collected in Surat Thani Province, Thailand. Compounds **204** and **206** both exhibited weak cytotoxic activity against KB cells, with IC_50_ values of 10.7 µM and 13.8 µM [48].

A known butanoic acid (**207**) was identified from the endophytic fungus *Xylaria feejeensis* strain AML-02 and isolated from *Avicennia marina* leaves collected at Wat Asokaram, Samut Prakan, Thailand [83]. A known kojic acid (1,5-hydroxy-2-hydroxymethyl-γ-pyrone) (**208**) was isolated from the endophytic fungus *Colletotrichum gloeosporioides* obtained from the leaves of *Sonneratia apetala* in the Sundarbans mangrove forest, Bangladesh [102]. Compound **208** exhibited significant antimicrobial activity, with the highest antibacterial activity against *Micrococcus luteus* (MIC = 7.03 × 10^2^ µM), and the weak activity against *Pseudomonas aeruginosa* (MIC = 8.79 × 10^2^ µM). The ethyl acetate extract of the fungus demonstrated even greater potency, particularly against *Pseudomonas aeruginosa* (MIC = 1.69 µM), comparable to the positive control, ciprofloxacin (MIC = 8.45 × 10^−1^ µM) [102] (Figure 13).

### 2.2. Terpenoid Derivatives

Terpenes and terpenoids belong to the biggest class of secondary metabolites and natural products, and basically consist of five carbon units, called isoprene units [103]. These monomeric isoprene units join each other in many ways to produce structurally diverse terpenes. More than 25,000 terpenoids have been reported [104]. These terpenes are categorized based on the number of isoprene units combined together. Terpenes derivatives have been identified from mangrove-associated fungi and are observed to possess some biological activities, including anti-microbial, anti-malarial, anti-cancer, antitumor, etc. [89]. Moreover, terpenes are used in the food industry and fragrance industry, in addition to their use in medicinal applications and as a precursor for the synthesis of medicinal drugs. Fungal terpenoids are of great interest due to their biological properties [105,106,107]. A total of **60** terpenoids are documented in this paper as fungal metabolites, which include **47** new compounds, and **29** compounds with bioactivity were found.

**Figure 14:**
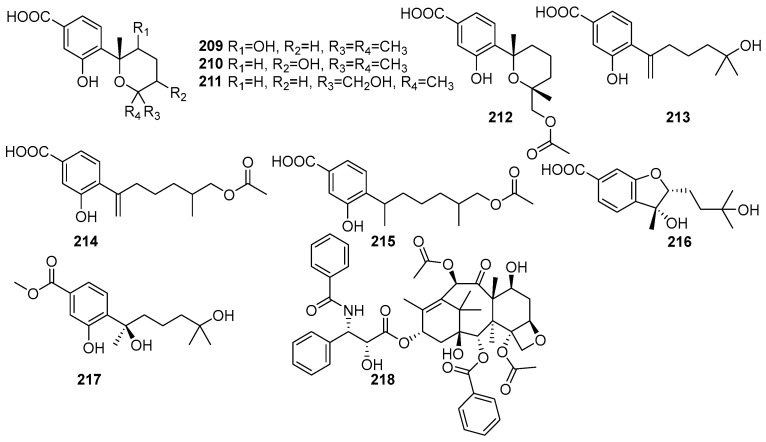
Meroterpenoid derivatives in terpenoids isolated from mangrove fungi (**209**–**218**).

Meroterpenes are characterized by their mixed biosynthetic origin, consisting of both terpenoid and polyketide moieties. The monoterpenoid derivatives isolated from mangrove fungi are shown in Figure 14. They are complex natural products. Eight new meroterpenes, (7R,8R)-8-hydroxysydowic acid (**209**), (7S,10S)-10-hydroxy-sydowic acid (**210**), (7S,11R)-12-hydroxy-sydowic acid (**211**), (7S,11R)-12-acetoxy-sydowic acid (**212**), 7-deoxy-7,14-didehydro-11-hydroxysydonic acid (**213**), (7-deoxy-7,14-didehydro-12-acetoxy-sydonic acid (**214**), (E)-7-deoxy-7,8-didehydro-12-acetoxy-sydonic acid (**215**), (7R,8R)-1,8-epoxy-11-hydroxy-sydonic acid (**216**), and (7R)-11-hydroxy-sydonic acid methyl ester (**217**) were isolated from the endophytic fungus *Aspergillus versicolor* obtained from the mangrove plant *Avicennia marina* in the Red Sea, Egypt [84]. These compounds were evaluated for their cytotoxic activity against HeLa cells, and their IC_50_ values were found to be **209** (IC_50_ = 43.7 μM), **214** (IC_50_ = 83.8 μM), **213** (IC_50_ = 83.8 μM), and **215** (IC_50_ = 53.5 μM). Compound **209** exhibited moderate antimicrobial activity against Gram-positive bacteria, while **210** showed weak antimicrobial activity. Compound **211** demonstrated cytotoxicity with an IC_50_ value of 83.8 μM, while **214** and **215** showed weak cytotoxic activity. Compound **217** (ergosterol peroxide) showed weak antimicrobial and cytotoxic activity [84]. A tetracyclic diterpene lactam, taxol (**218**), was isolated from the endophytic fungus *Fusarium oxysporum* obtained from the leaves of *Rhizophora annamalayana* in the Vellar estuary, Tamil Nadu, India. Compound **218** was identified as having potent cytotoxic activity against KB and KBV200 cells (IC_50_ < 58.6 µM) [108].

**Figure 15:**
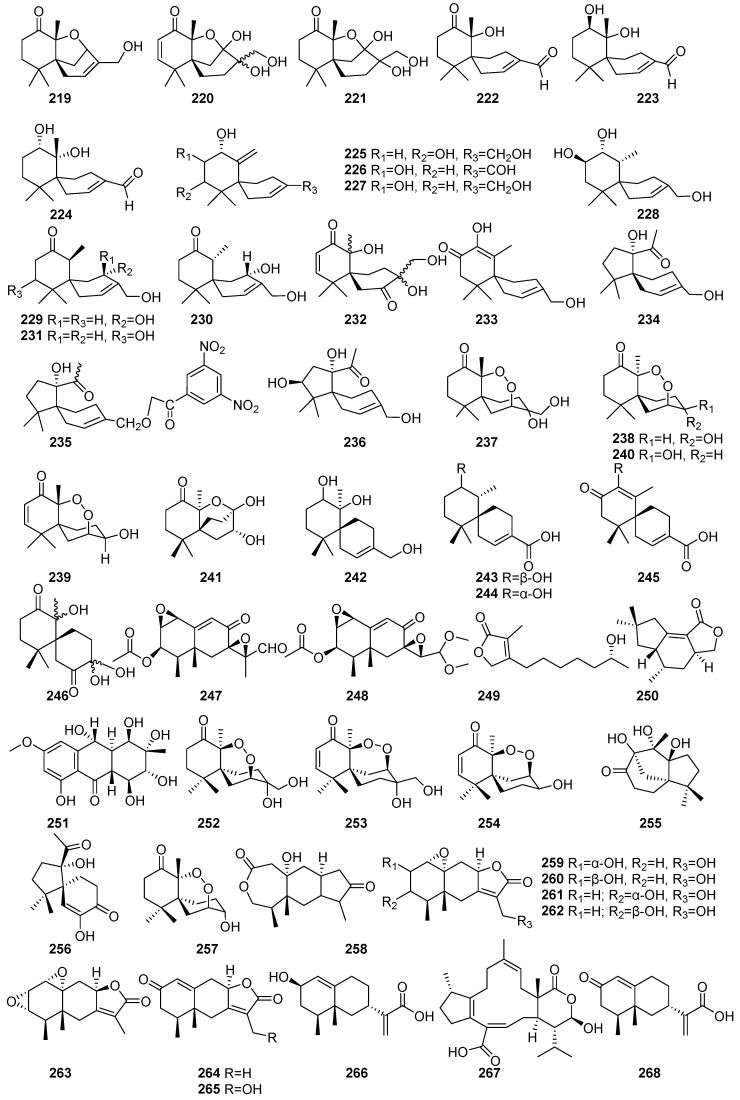
Sesquiterpenes and monoterpenes in terpenoids isolated from mangrove fungi (**219**–**268**).

Figure 15 (**219**–**268**) shows some sesquiterpenoid bioactive compounds isolated from mangrove fungi. Twenty novel sesquiterpenoid compounds (**219**–**238**) and two known endoperoxides (**253** and **254**) were isolated from the endophytic fungus *Pseudolagarobasidium acaciicola* obtained from the mangrove tree *Bruguiera gymnorrhiza* collected in Thailand. The scientist [109] has identified several Acaciicolides from fungal metabolites. Acaciicolide A (**219**), B (**220**), C (**221**), and Acaciicolinols A–L (**222**–**233**) were characterized with a 6/6 spirobicyclic ring system. Among them, **223** displayed weak activity against MOLT-3 (IC_50_ = 165.04 μM) and HL-60 (IC_50_ = 159.05 μM) [109]. Another study found that compounds, including acaciicolinols C (**224**), D (**225**), F (**227**), and K (**232**), were isolated from the mangrove-derived endophytic fungus XG8D obtained from the leaves of *Xylocarpus granatum* collected in Thailand [110]. These known chamigrane sesquiterpenes (**224**–**227** and **232**) demonstrated cytotoxic activity against MCF-7, Hep-G2, and KATO-3 cells at 50 µM [110]. Spiroacaciicolides B and C (**234** and **235**), which featured a unique 5/6 spirobicyclic system, were not subjected to cytotoxic evaluation [109].

Merulin-type endoperoxides (**236**–**240**) were demonstrated to have significant cytotoxic potential. 7-epi-TMC-264B (**236**) exhibited potent and selective activity against HL-60 (IC_50_ = 0.28 μM; SI = 64.0). Compound **237**, 3-epi-merulin A, demonstrated moderate cytotoxicity, with IC_50_ values of 12.09–170.08 μM across cancer cell lines. The known compound (**136**) and bioactive merulins A (**239**) and D (**240**) exhibited moderate and selective cytotoxicity, respectively [109]. Compound **244** was isolated from the endophytic basidiomycetous fungus XG8D, derived from the mangrove plant *Xylocarpus granatum* collected in Samutsakorn Province, Thailand. These compounds represent rare chamigrane-type endoperoxides. Compound **239** exhibited moderate cytotoxic activity against BT474 and SW620 cancer cell lines, with IC_50_ values of 19.0 µM and 19.6 µM, respectively [111]. Further, it is also found that **239** and **240** were isolated from the endophytic fungus XG8D derived from the mangrove plant *Xylocarpus granatum* collected in Samutsakorn Province, Thailand [112]. Six novel compounds, including merulinols A–F (**241**–**246**), were isolated from the mangrove-derived endophytic fungus XG8D obtained from the leaves of *Xylocarpus granatum* collected in Thailand. Merulinols C and D (**243** and **244**) selectively inhibited KATO-3 cells (IC_50_ = 35.0 and 25.3 µM) [110] (Figure 15).

Known compounds, including PR-Toxin (**247**) and penicilliumolides F (**248**), were isolated from the mangrove endophytic fungus *Penicillium chermesinum* strain HLit-ROR2 obtained from the leaves of *Xylocarpus granatum* collected at the Mangrove Forest Learning and Development Center 2, Samut Sakhon province, Thailand. These compounds exhibited moderate cytotoxic activity against T47D, MDA-MB-231, HepG2, and MOLT-3 cell lines [56]. A known sesquiterpene lactone compound namely, seiridin (**249**), was isolated from the *Pestalotiopsis* sp. PSU-MA69, an endophytic fungus obtained from *Rhizophora apiculate,* collected in Thailand [68]. Tremulenolide A (**250**) was isolated from *Flavodon flavus* PSU-MA201 obtained from the leaf of *Rhizophora apiculata* collected in Thailand [99]. Originally, it was reported from *Phellinus tremulae,* and this compound exhibited moderate antibacterial and antifungal activities, each with MIC values of 484.3 µM against *Staphylococcus aureus* ATCC 25923 and *Cryptococcus neoformans* ATCC 90113, respectively [99]. A known ampelanol (**251**) was isolated from the fermentation extract of the mangrove-derived fungus *Phomopsis* sp. PSU-MA214 collected from the leaves of *Rhizophora apiculata* in Songkhla Province, Thailand. Compound **251** exhibited weak cytotoxic activity against both MCF-7 cancer cell lines, with IC_50_ values > 146.9 µM [52] (Figure 15).

The new merulins, B (**252**) and C (**253**), were isolated from the endophytic basidiomycetous fungus XG8D derived from the mangrove plant *Xylocarpus granatum* collected in Samutsakorn Province, Thailand. They represent rare chamigrane-type endoperoxides. Compound **253** displayed stronger cytotoxic effects, with IC_50_ values of 5.5 µM (BT474) and 14.5 µM (SW620) [111]. Another study also found **252**, **253**, and stepperoxide A (**254**) were isolated from the endophytic fungus XG8D derived from the mangrove plant *Xylocarpus granatum* collected in Samutsakorn Province, Thailand. Compound **253** exhibited potent antiangiogenic activity, completely inhibiting micro vessel sprouting in the rat aortic (ex vivo) ring assay at 2.5 μM and suppressing neovascularization in the in vivo mouse Matrigel plug assay at 10 μM. It also strongly inhibited HUVEC proliferation (IC_50_ = 0.9 μM), VEGF-induced migration, and tube formation, likely via suppression of the Erk1/2 signaling pathway without affecting Akt phosphorylation. In contrast, **254** showed weaker antiangiogenic activity, with complete inhibition at 25 μM [112]. Further, **252**–**254** were also isolated from the endophytic fungus *Pseudolagarobasidium acaciicola* obtained from *Bruguiera gymnorrhiza* collected in Thailand. Compound **252** exhibited weak cytotoxicity against MOLT-3 and HepG2 (IC_50_ = 44.2 and 181.6 µM). Compound **253** showed strong cytotoxic activity against the MB-231 and HL-60 cell lines, with IC_50_ values of 0.28 and 13.18 µM, while **254** showed similar cytotoxic potency, with IC_50_ values of 2.7 and 21.3 µM [113]. Three novel compounds, acaicolin A (**255**), spiroacaciicolide A (**256**), and 3-epi-steperoxide A (**257**), were isolated from the endophytic fungus *Pseudolagarobasidium acaciicola* obtained from *Bruguiera gymnorrhiza* collected in Thailand. Compound **257** showed high cytotoxicity across the tested cancer cell lines HuCCA-1 and MDA-MB-231, with IC_50_ values of 2.7 and 14.6 µM, respectively [113]. The known compounds **256** and **261**–**265** and several novel compounds, namely spiroacaciicolide A (**257**), eremophilanolides A (**258**), B (**259**), C (**260**), xylareremophil (**261)**, xylarremophil A (**262**), crotonepoxide (**263**), 7-hydroxyxylareromophil (**264**), 13-hydroxy-xylareremophil A (**265**), and eremophilane (**266**), were isolated from the mangrove-derived fungus *Xylariaceae* sp. BCC 60405 obtained from mangrove wood collected at Ko Hua Ta Chio, Trat Province, Thailand [64]. Compound **257** showed weak cytotoxicity against Vero cells, with an IC_50_ value of 119.1 µM. Compound **261** displayed antibacterial activity against *Micrococcus luteus* (MIC = 114.5 µM) [64]. An unusual tricyclic sesterterpene, bipolarenic acid (**267**), was isolated from the marine-derived fungus *Lophiostoma bipolare* BCC25910, collected from mangrove wood in Haad Wanakorn National Park, Thailand [69]. A new compound, 7-epi-tessaric acid (**268**), was isolated from the mangrove-derived fungus *Xylariaceae* sp. BCC 60405 obtained from mangrove wood collected at Ko Hua Ta Chio, Trat Province, Thailand [70] (Figure 15).

### 2.3. Alkaloid Derivatives

Alkaloids are nitrogen-containing natural products produced by plants, microorganisms, and marine organisms with potent biological activity. Alkaloids have diverse and important physiological effects on humans and other animals [114,115]. Alkaloids are N-containing organic compounds. There is a large diversity of alkaloids, and all can be categorized into two groups. In typical alkaloids, one or more nitrogen atoms are present as ring atoms, whereas atypical alkaloids, or alkaloid amine, contain nitrogen that is not in the ring system [116].

**Figure 16:**
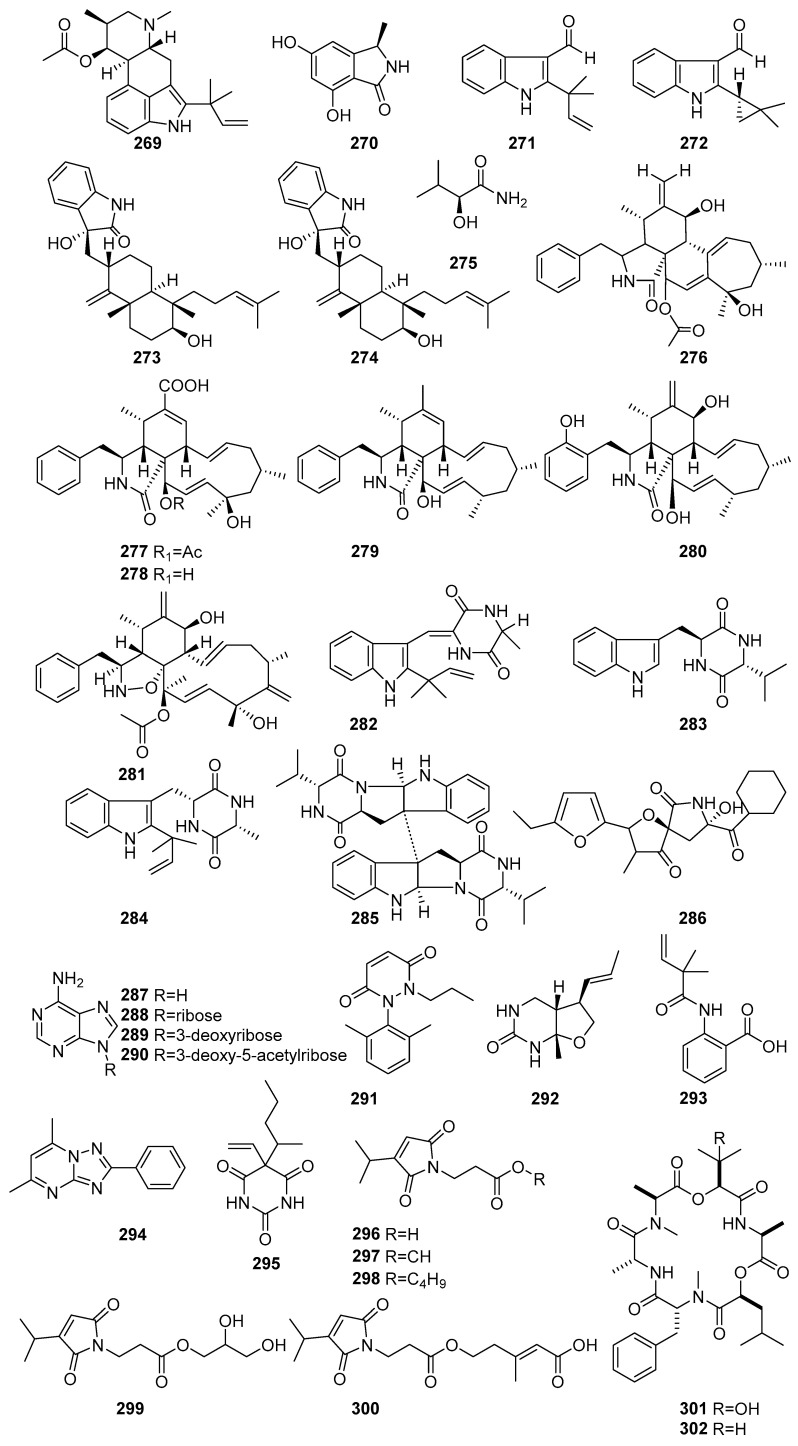
Derivatives of alkaloids isolated from mangrove fungi (**269**–**302**).

Figure 16 (**269**–**302**) shows the derivatives of alkaloids isolated from mangrove fungi. Fumigaclavine C (**269**), a known indole-diterpene alkaloid, was isolated from the endophytic fungus *Aspergillus fumigatus* obtained from the leaves of *Ceriops decandra*, a mangrove plant collected from the Sundarbans mangrove forest, Bangladesh. Compound **269** exhibited antibacterial activity against *Pseudomonas aeruginosa* (MIC = 425.8 µM) and *Micrococcus luteus* (MIC = 212.9 µM) [91]. A novel iso-indolinone derivative, (3R)-5,7-Dihydroxy-3-methylisoindolin-1-one (**270**), was isolated from the endophytic fungus obtained from the mangrove plant *Avicennia marina* collected from Oman. Compound **270** exhibited weak antibacterial activity against *Streptococcus pneumoniae* at 331.3 µM [112]. Two new compounds, namely 2-(2-methyl-3-en-2-yl)-1H-indole-3-carbaldehyde (**271**) and 2-(2,2-dimethylcyclopropyl)-1H-indole-3-carbaldehyde (**272**), were isolated from the fermentation extract of the mangrove-derived endophytic fungus *Eurotium chevalieri* KUFA 0006 obtained from *Rhizophora mucronata* collected in Thailand. Compound **271** displayed moderate antibacterial activity (MIC = 300.1 µM) against *S. aureus* ATCC 25923 [51]. Two new compounds, Anthcolorins G (**273**) and H (**274**), were isolated from the mangrove-derived endophytic fungus *Aspergillus versicolor* obtained from the fruits of *Avicennia marina* in the Red Sea, Egypt. Compound **274** showed cytotoxic activity against HeLa cells, with an IC50 value of 99.8 µM [84] (Figure 16). A known amide derivative, **275**, aliphatic amide, was isolated from the mangrove-derived fungus *Phomopsis* sp. PSU-MA214 obtained from the leaves of *Rhizophora apiculata* collected in Songkhla Province, Thailand [52].

Cytochalasins are a class of fungal metabolites known to be present in endophytic fungi. Those are reported to possess a wide range of biological activities, making them promising candidates for various therapeutic applications. There are six classes of cytochalasin biosynthesized from an amino acid. A novel cytochalasin named cytochalasin H (**276**) was isolated from the endophytic fungus *Diaporthe amygdali* SgKB4 obtained from the bark of *Sonneratia griffithii* in the mangrove ecosystem of Bungus Coast, Padang, West Sumatra, Indonesia [117]. Four novel cytochalasin compounds, including phomopsichalasins D-G (**277**–**280**), were isolated from the mangrove endophytic fungus *Phomopsis* sp. xy21 obtained from the leaves of the mangrove plant *Xylocarpus granatum* collected in Trang Province, Thailand. Phomopsichalasin E (**278**) showed moderate cytotoxicity against several of these cell lines, with IC_50_ values of 42.9 µM and 87.4 µM against HCT-8 and A2780 cancer cell lines. Phomopsichalasin F (**279**) demonstrated moderate-to-potent cytotoxic effects, notably against HCT-8/T (IC_50_ = 11.2 µM) and A2780 (IC_50_ = 8.6 µM). The most active compound, phomopsichalasin G (**280**), exhibited strong cytotoxicity against HCT-8 (IC_50_ = 7.5 µM), A549 (IC_50_ = 6.4 µM), and MDA-MB-231 (IC_50_ = 3.4 µM) [118]. Known cytochalasin D (**281**) was isolated from the mangrove-derived fungus *Xylaria cubensis* PSU-MA34 obtained from a branch of *Bruguiera parviflora* collected in Surat Thani Province, Thailand. Compound **281** exhibited weak cytotoxic activity against KB cells, with an IC_50_ value of 7.9 µM [48] (Figure 16).

These compounds possess a stable six-membered ring, which is an important pharmacophore. Some bioactive diketopiperazines have been reported from fungi, including endophytic fungi. A novel diketopiperazine derivative, (3,1′-Didehydro-3-[2″-(3″,3″′-dimethyl-prop-2-enyl)-3″′-indolylmethylene]-6-methylpiperazine-2,5-dione) (**282**), was isolated from the endophytic fungus *Penicillium chrysogenum* MTCC 5108 obtained from the mangrove plant *Porteresia coarctata* in Goa, India [119]. Compound **282** exhibited significant antibacterial activity against *Vibrio cholerae,* with a MIC value of 49.5 µM, comparable to the standard antibiotic streptomycin (MIC = 24.7 µM) [119]. Known compounds, including (11S, 14R)-cyclo(tryptophylvalyl) (**283**), echinulin (**284**), and eurocristatine (**285**), were isolated from the mangrove-derived endophytic fungus *Eurotium chevalieri* KUFA 0006 obtained from *Rhizophora mucronata* collected in Thailand [51]. The known cyclic peptide, azaspirofuran B (**286**), was isolated from *Aspergillus fumigatus*, a mangrove-derived endophytic fungus from *Ceriops decandra* collected from the Sundarbans mangrove forest, Bangladesh. Compound **286** demonstrated moderate antibacterial activity, with a MIC value of 96.2 µM against *S. aureus* [91] (Figure 16). 

Known compounds, including adenine (**287**), adenosine (**288**), 3′-deoxyadenosine (**289**), and 3′-deoxy-5′-acetyladenosine (**290**), were isolated from the mangrove rhizosphere-associated fungus *Emericella* sp. strain SWR1718 obtained from the rhizosphere soil of *Avicennia marina* collected from the Jeddah coastline, Saudi Arabia [50]. Compound **288**, a purine nucleoside, showed weak cytotoxicity against HTB-176 (IC_50_ = 42.9 µM). In contrast, **289**, a purine nucleoside derivative, exhibited weak cytotoxicity across all three tested cell lines, with IC_50_ values of 40.7 µM (HTB-176), 77.8 µM (SW-620), and 75.2 µM (HT-29). 

The new pyridazine derivatives, 1-(2′,6′-Dimethylphenyl)-2-n-propyl-1,2-dihydropyridazine-3,6-dione (**291**), were isolated from the mangrove-derived endophytic fungus *Aspergillus* sp. AV-2 obtained from the leaves of *Avicennia marina* in Hurghada, Red Sea, Egypt [56]. Compound **291** exhibited moderate cytotoxic activity against Caco-2 cells, with an IC_50_ value of 18.2 μM, and represents the first natural occurrence of this phenyl pyridazine scaffold [56]. Astronyurea (**292**), a new cyclic urea derivative, was isolated from the mangrove-associated fungus *Astrosphaeriella nypae* BCC 5335 obtained from the mangrove palm *Nypa fruticans* collected from Samut Prakan Province, Thailand [55]. Compound **292** exhibited moderate antibacterial activity against *Staphylococcus aureus* (MIC = 159.8 µM) and *Escherichia coli* (MIC = 228.2 µM), along with weak cytotoxicity against Vero cells (IC_50_ = 105.9 µM) [55].

A novel anthranilic acid derivative, 2-[(2,2-dimethylbut-3-enoyl) amino] benzoic acid (**293**), was isolated from the mangrove-derived endophytic fungus *Eurotium chevalieri* KUFA 0006 obtained from *Rhizophora mucronata* collected in Thailand. Compound **293** exhibited weak antibacterial activity against *Enterococcus faecalis* ATCC 29212 (MIC = 274.4 µM) [46]. The known compounds 1,2,4-triazolo [1,5-a] pyrimidine, 5,7-dimethyl-2-phenyl (**294**), and vinylbital (**295**) were isolated from the *Actinomycetes* strain SMS_SU21 collected from the Sundarbans mangrove forest, Bangladesh. Compound **294** exhibited potent antifungal activity, particularly against *Rhizoctonia solani* and *Macrophomina phaseolina* (MIC value of 209.8 µM), and **294** showed significant antibacterial activity, including against *Escherichia coli*, with an MIC value of 210 µM. Compound **295** demonstrated significant inhibition against *Vibrio cholerae* and *Staphylococcus aureus*, with MIC values of 167.9 µM [80]. The known farinomalein derivatives, farinomaleins A–E (**296**–**300**), were isolated from the endophytic fungus obtained from the mangrove plant *Avicennia marina* collected from Oman. Compounds **297** showed moderate cytotoxicity, with an IC_50_ value of 20.8 µM against the lymphoma cell (L5178Y) line [112] (Figure 16). Two cyclodepsipeptides, guanomides A (**301)** and B (**302**), were isolated from Acremonium sp. PSU-MA70, a branch of mangrove *Rhizophora apiculata*, collected in Satun Province, Thailand, with their bioactivities not reported [72]. Alkaloids, secondary metabolites produced in mangrove fungi, are complex compounds with a range of bioactivities, with predominant antibacterial activity. This shows the alkaloid derivatives to be developed as novel antibiotics, which would be a better alternative due to prevalent antibiotic resistance.

## 3. A Comprehensive Evaluation of Current Research Efforts: Tissue Preference and Geographical Imbalance in Mangrove Fungal Studies

**Figure 17:**
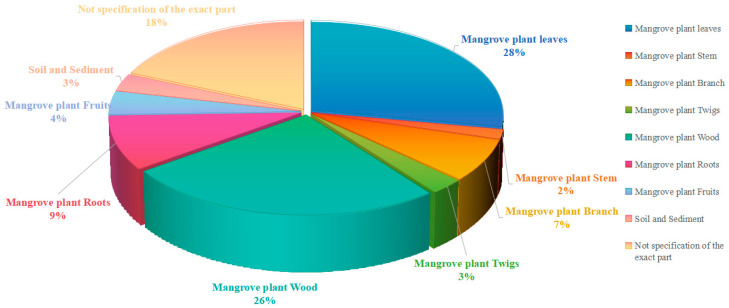
Distribution of mangrove fungal samples by tissue origin.

**Figure 18:**
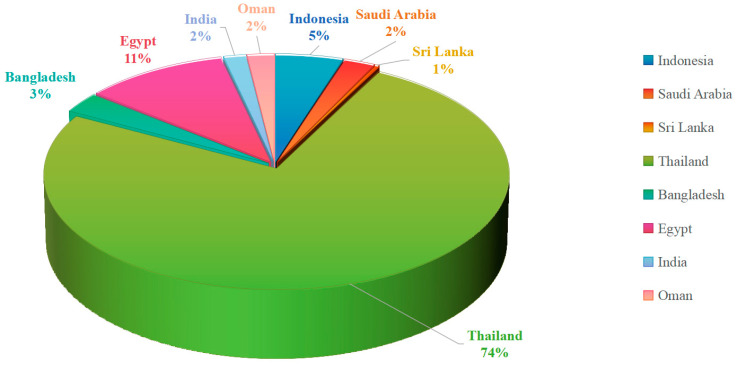
Geographical distribution of mangrove fungal studies along the Indian Ocean coast.

Despite the recognized pharmaceutical potential of mangrove fungi, the allocation of research efforts—*where* to look for them and *what* to isolate them from—has not been systematically evaluated in the review. To uncover potential biases and guide future exploration, this section provides a critical analysis of two fundamental aspects of the current research: the source of plant tissues/environmental samples used for fungal isolation and the geographical distribution of these studies (Table S1). A systematic review of the **302** compounds included here reveals significant tissue preference and a pronounced geographical imbalance, as clearly illustrated in the following figures (Figure 17 and Figure 18).

The statistical analysis of the tissue origins of mangrove fungi in the existing literature reveals a distinct sampling bias. The data shows that most studies have focused on wood and leaves, which account for a significant proportion of the samples (ex, [53,60,69,70,73,74,75]). In contrast, other tissues, such as fruits, twigs, and crucially, the sediment around roots, are severely under-represented.

This unbalanced sampling pattern likely leads to a biased understanding of the mangrove fungal community and its metabolic potential. Wood, as a rich source of lignin and cellulose, has long been considered an ideal habitat for lignicolous fungi, which explains why fungi isolated from this source often produces a wealth of bioactive secondary metabolites [120]. However, the neglect of fruit represents a significantly missed opportunity. As the reproductive organs of the plant, fruits are nutrient-rich and may harbor highly specialized fungal communities that produce unique metabolites to protect seeds or attract dispersers, making them a valuable direction for future research.

An analysis of the geographical origins of the studies included in this review reveals a strikingly uneven distribution. Of the **302** compounds shown in this review, Thailand alone contributed **227**, accounting for an overwhelming majority (approximately **75%**). While other regions along the Indian Ocean coast, such as Indonesia, Egypt, and Saudi Arabia, have some research output, their numbers are far lower. Meanwhile, countries like India and Sri Lanka are represented by only a handful of studies, indicating that they are largely unexplored frontiers.

This high degree of geographical concentration implies that our current understanding of “Indian Ocean mangrove fungi” is, in essence, primarily an understanding of “Thai mangrove fungi.” Thailand’s pre-eminence in this field is likely attributable to its well-established scientific infrastructure, long-standing research interest, and its extensive and diverse mangrove ecosystems.

Therefore, one of the most urgent directions for future research is the systematic filling of these vast geographical gaps. Under-explored regions such as India, Sri Lanka, Bangladesh, and the East African coast should be prioritized. Such systematic exploration will not only lead to the discovery of new species and compounds but, more importantly, will allow us to understand, from a comparative metagenomic and environmental metabolomic perspective, how environmental factors drive the evolution and distribution of fungal secondary metabolites. In conclusion, only through geographically balanced and systematic efforts can we truly unveil the full potential of the Indian Ocean coast’s mangrove fungi as a treasure trove for drug discovery.

## 4. Contributions of Mangrove-Associated Fungal Metabolites to Pharmaceutical, Drug Discovery, and Industrial Applications

This review provides comprehensive research data on how Indian coast mangrove-associated fungi are important, by serving as a bioactive reservoir that can be used in pharmaceutical and industrial applications. These microorganisms are a source for a variety of bioactive natural products, including antibiotics, anticancer agents, immunosuppressants, and agrochemicals, including biopesticides [37]. Therefore, fungi, particularly those mangrove-associated, are the primary source of novel bioactive compounds. As described above, numerous novel metabolites were isolated from mangrove-associated microbial strains [121,122]. It was estimated that 850 novel bioactive compounds have been investigated from mangrove-derived fungi, possibly for drug discovery [123]. About 2603 novel compounds were documented by [124] to elucidate their chemical structures, bioactivity, geographic origins, and the taxonomic classification of the source organisms. Based on all the information reported in this review, it is obvious that mangrove-associated fungi from the Indian Ocean coastline are a promising source of chemically novel and biologically effective compounds with both medicinal and industrial scale applications [44,125,126,127]. This is attributed to the extreme and unique environmental pressures in the Indian Ocean [128].

Marine biotechnology has enabled the production of advanced pharmaceutical products, including wound-healing materials such as injectable hydrogels and topical dressings, from marine-derived polysaccharides and chitosan [129,130]. Endophytic fungi isolated from Malaysian mangroves, particularly *Lasiodiplodia theobromae* and *Fusarium* sp., produce diverse isocoumarins and naphthoquinones with effective anti-trypanosoma brucei activity, offering promising leads for novel anti-trypanosomal agents that target chemotherapeutics and as adjunctive therapies for human sleeping sickness [131]. Furthermore, an endophytic strain of *Aspergillus fumigatus* cultured from mangrove tissues collected in the Sundarbans, Bangladesh, was found to be the source for azaspirofuran B and used as a potential compound for the invention of novel antibacterial agents against drug-resistant pathogens [91]. Similarly, mangrove-derived fungi and actinomycetes yield peptides, alkaloids, and polyketides, exhibiting antimicrobial activity against broad human pathogens. In addition, they are significant for anticancer and anti-inflammatory agents originating from mangroves [132]. The fungi *Schizophyllum commune* isolated from Indian mangroves produces bioactive polysaccharides and terpenoids with antitumor and immunomodulatory effects [129,133]. The fungus *Aspergillus ustus*, from Thai mangroves, isolated ophiobolins used against renal carcinoma cells; several of the isolated ophiobolins show antiproliferative activity [134].

Beyond therapeutics, mangrove bioprospecting in the Indian Ocean has become an emerging frontier for cosmeceutical innovations. Bioactive compounds isolated from mangrove-associated microorganisms and plants are increasingly applied in cosmetic product development due to their antioxidants, photoprotective, anti-aging, and skin-regenerative properties [135]. Recently reported thraustochytrid strains are potent in producing DHA-rich oils and carotenoids with notable antioxidant activity, highlighting their potential use in skin-care formulations [136,137]. A previous study confirmed their ecological abundance and applicability in large-scale metabolite production [32]. Their carotenoid profiles, including astaxanthin, further highlight their possibility as candidates for anti-ageing and UV-protective formulations [138]. The secondary metabolites produced by these fungi have radical-scavenging, melanin-suppressing, and tyrosinase-inhibiting properties, which are useful for developing skin-whitening and anti-wrinkle formulations [139]. The melanin precursor from *Aspergillus nidulans* SG 28 exhibits antioxidant, UV-filtering (SPF 9.9), and cytocompatibility activity, demonstrating its application in skincare and anti-ageing product formulation [140]. Collectively, these insights highlight the growing role of mangrove-derived microbial metabolites in sustainable cosmeceutical development across the Indian Ocean region.

Although mangrove-derived fungi exhibit remarkable chemical diversity and pharmacological potential, their conversion into applications of pharmaceutical and industrial products remains constrained by significant challenges in the Indian Ocean region. This disparity highlights the need to identify untapped opportunities, address key bottlenecks, and overcome barriers that hinder the development of scalable innovations. These challenges, together with potential future directions, are explored in the following section.

## 5. Challenges and Future Directions in the Discovery and Applications of Natural Products from Indian Ocean Mangrove Fungi

Although there are significant number of bioactive secondary metabolites that have been identified from Indian Coast marine-associated fungi, this ecosystem is yet to be explored in full strength to discover novel bioactives [141]. The exploration of natural products from mangrove-associated fungi in the Indian Ocean region faces numerous scientific, technological, ecological, and infrastructural barriers that have hindered progress in this promising, yet underdeveloped, field [142,143]. One key issue is the ambiguity surrounding the classification of “marine-derived” fungi, especially those isolated from mangrove tissues that are not consistently submerged or exposed to high salinity [144,145,146].

Numerous challenges are being faced in isolating fungal metabolites from mangrove-associated fungi using conventional methods, such as cultivating such fungi under laboratory conditions, requiring much concern for providing for the natural environment [141]. Some strains grow extremely slowly or fail to sporulate, and the absence of in situ host signals often leads to the suppression of their unique metabolite biosynthesis. This is further compounded by the widespread presence of silent biosynthetic gene clusters (BGCs), which remain inactive or “silent” under standard culture conditions [147]. Techniques such as the One-Strain Many Compounds (OSMAC) approach, co-culturing, and epigenetic modulation have been used to stimulate expression, but their application requires significant technical skill and resource investment [141,148]. Despite the availability of cutting-edge omics technologies such as metabolomics, transcriptomics, and genome mining, full integration of such technologies into mangrove mycological research is hindered by bioinformatics challenges, a lack of expertise in the field, and insufficient data-sharing networks in the region.

However, continued effort on the discovery of bioactive secondary metabolites from mangrove fungi will result in chemical compounds with potent antibacterial (including activity against MRSA and drug-resistant TB), anticancer, anti-inflammatory, antioxidant, antidiabetic, and immunosuppressive properties, which can be developed into novel drugs [145,149]. Moreover, natural products from mangrove endophytes have shown potential as selective inhibitors of critical disease targets, such as protein tyrosine phosphatases and kinases.

Advanced “Omics” technologies paired with high-throughput tools and powerful bioinformatics platforms enable the identification of biosynthetic gene clusters (BGCs), the prediction of metabolite pathways, and the discovery of non-cultivable fungal species that are biologically and industrially important [145,147,148].

Mangrove fungi are also a source of novel enzymes [150], and it has been reported that many fungi can produce enzymes with physiological characteristics suited to extreme conditions like varying temperature, pressure, pH, and salinity, for applications in animal feed, bread-making, juice and wine industries, and xylitol production [146]. Though potential industrial uses are possible for such enzymes, industrial-scale production of such enzymes has not yet been realized. Some examples of synthesis of industrially important enzymes are *Aspergillus niger*, which is a source for thermostable xylanase [151] that can be used in paper pulp bioleaching, and marine fungus NIOCC#2a, which is a source for laccase that can be used for bioremediation of colored effluents and synthetic dyes [152]. A hypersaline-tolerant white-rot fungus, *Phlebia* sp. MG-60, shows potential for lignin degradation, which is useful in producing animal feed from sugarcane bagasse [146,153].

Moreover, the ecological roles of marine fungi, including nutrient cycling, organic matter decomposition, and environmental detoxification, highlight their potential in bioremediation, including the degradation of persistent pollutants like textile effluents and polycyclic aromatic hydrocarbons [145], and uses as biopesticides and biocontrol agents in sustainable agriculture.

It can be emphasized that future efforts related to mangrove fungi research should prioritize the development of national databases and fungal repositories and the expansion of marine mycology research infrastructure. Integration of fungal research with conservation initiatives will be crucial for preserving the ecological functions of mangrove ecosystems while harnessing their biotechnological potential. Importantly, interdisciplinary collaborations among mycologists, chemists, molecular biologists, environmental scientists, and engineers, as well as public–private partnerships, are essential to translating marine fungal metabolites and enzymes into real-world applications. Such integrated and collaborative approaches will pave the way for innovations in drug discovery, industrial biotechnology, and sustainable environmental remediation. 

## 6. Conclusions

This review synthesizes research on structurally diverse secondary metabolites identified from Indian Ocean mangroves’ endophytic fungi, along with their pharmacological activities covering the reporting period of 2002–2025. About **302** compounds are reported in this review, including **164** novel chemical compounds with notable bioactivities such as cytotoxicity, antibacterial, antifungal, antioxidant, anti-inflammatory, and *α*-glucosidase inhibitory effects. These compounds mainly belong to the classes of polyketides (**208** compounds), alkaloids (**34** compounds), and terpenoids (**60** compounds). Polyketide compounds are reported to possess cytotoxicity as the dominant bioactivity, whereas terpenoids and alkaloids are reported as dominant antimicrobial activities out of many activities shown. These findings highlight the significant potential of these fungi in pharmaceutical, agricultural, and industrial biotechnology. However, research remains geographically uneven, with African, Middle Eastern, and Indian mangrove regions underrepresented. Most studies are limited to in vitro screening, lacking mechanistic, in vivo, and structure–activity analyses. Future efforts should integrate genomics, metabolomics, and synthetic biology to activate silent biosynthetic pathways and explore untapped chemical diversity. Sustainable bioprospecting and interdisciplinary collaboration are essential to translating these resources into therapeutics and industrial applications. By addressing these gaps, Indian Ocean mangrove fungi can become a cornerstone for biotechnological innovation.

## Figures and Tables

**Table 1:** Number of mangrove species in the Indian Ocean coastal countries.

Country (Indian Ocean)	No. of Species	Reference
Indonesia	48	[15,16]
India	39	[17,18]
Malaysia	38	[19,20]
Australia	41	[21,22]
Thailand	33	[23,24]
Myanmar	31	[20]
Bangladesh	27	[24,25]
Sri Lanka	23	[26,27]
Maldives	15	[28]
Mozambique	10	[29,30]
Madagascar	8	[31]
Kenya	9	[32]
Tanzania	9	[32,33]
Oman	2	[34]
Iran	2	[35,36]

## Data Availability

Data are contained within the article and Supplementary Materials.

## Supplementary Materials

The following supporting information can be downloaded at https://www.mdpi.com/article/10.3390/molecules31020261/s1, Table S1: Information of natural products (**1**–**302**) from Mangrove-Associated Fungi along the Indian Ocean Coast.
