# Supplementary material for: Advances in Natural Products from Mangrove-Associated Fungi Along the Indian Ocean Coast
Source: Molecules. 2026 Jan 12;31(2):261. doi: 10.3390/molecules31020261 (PMC12843673; doi:10.3390/molecules31020261)
Supplement: Supplementary file 1 [file molecules-31-00261-s001.zip › molecules-4041288-supplementary.pdf]

Table S1. Information of natural products (**1-302**) from Mangrove-Associated Fungi along the Indian Ocean Coast.

Compounds	Mangrove Species	Fungal Strains	Region/ Country	Bioactivities	
2-chloro-5-methoxy-3-methylcyclohexa-2,5- diene-1,4-dione **(1)**	*Bruguiera parviflora* (Branches)	*Xylaria cubensis* PSU-MA34	Surat Thani Province, Thailand		[48]
6-hydroxy-astropaquinone B **(2)** Novel	*Rhizophora mucronata* (Stem)	*Fusarium napiforme*	Makassar, South Sulawesi, Indonesia	Antibacterial: MIC (*Staphylococcus aureus* NBRC 13276) 20.8 μM, MIC (*P. aeruginosa* ATCC 15442) 20.8 μM.	[49]
Astropaquinone D **(3)** Novel	*Rhizophora mucronata* (Stem)	*Fusarium napiforme*	Makassar, South Sulawesi, Indonesia	Antibacterial: MIC (*S. aureus* NBRC 13276) 41.3 μM.	[49]
3-O-methyl-9-O-methylfusarubin **(4)**	*Rhizophora mucronata* (Stem)	*Fusarium napiforme*	Makassar, South Sulawesi, Indonesia	Antibacterial: MIC (*S. aureus* NBRC 13276) 20.8 μM, MIC (*Pseudomonas aeruginosa* ATCC 15442) 20.8 μM.	[49]
Fusarubin **(5)**	*Rhizophora mucronate* (Stem)	*Fusarium napiforme*	Makassar, South Sulawesi, Indonesia	Antibacterial: MIC (*S. aureus* NBRC 13276) 65.3 μM.	[49]
Javanicin **(6)**	*Rhizophora mucronata* (Stem)	*Fusarium napiforme*	Makassar, South Sulawesi, Indonesia	Antibacterial: MIC (*S. aureus* NBRC 13276) 34.5 μM.	[49]
Asperthecin **(7)**	*Avicennia marina* (Rhizosphere soil)	*Emericella* sp. SWR1718	Jeddah coastline, Saudi Arabia	Cytotoxicity: IC_{50} (human lymphoma cell line HTB-176) 36.2 μM, IC_{50} (human colorectal adenocarcinoma cell line HT-29) 82.8 μM, IC_{50} (human colon cancer cell line SW-620) > 100 μM.	[50]
Emodin **(8)**	*Rhizophora mucronata* (Inner twigs)	*Eurotium chevalieri* KUFA 0006	Eastern Seaboard of Thailand	Cytotoxicity: IC_{50} (L5178Y mouse lymphoma cell line) 12.6 μM.	[51]
Questin **(9)**	*Rhizophora mucronata* (Inner twigs)	*Eurotium chevalieri* KUFA 0006	Eastern Seaboard of Thailand		[51]

Physcion **(10)**	*Rhizophora mucronata* (Inner twigs)	*Eurotium chevalieri* KUFA 0006	Eastern Seaboard of Thailand		[51]
Questinol **(11)**	*Rhizophora mucronata* (Inner twigs)	*Eurotium chevalieri* KUFA 0006	Eastern Seaboard of Thailand		[51]
Macrosporin **(12)**	*Avicennia marina*	*Stemphylium globuliferum*	Hurghada, Red Sea, Egypt	Antibacterial: MIC (*S. aureus* ATCC 25923) 93.5 μM, MIC (*Enterococcus faecalis* ATCC 29212) 187.0 μM.	[52]
	Rhizophora apiculata (Leaves)	*Stemphylium globuliferum*	Songkhla province, Thailand	Antibacterial: MIC (anti-*Escherichia coli*) 2.30 μM, MIC (anti-*V. parahemolyticus*) 5.0 μM, and MIC (anti-*S. albus*) 15 μM.	[52]
	Avicennia marina	*Stemphylium globuliferum*	Hurghada, Red Sea, Egypt	Cytotoxicity: IC_{50} (L5178Y mouse lymphoma cell line) 7.9 μM.	[52]
1-Hydroxy-3-methoxy-6-methylanthraquinone **(13)**		*Phomopsis* sp. PSU- MA214	Songkhla province, Thailand		[49]
Acetylquestinol **(14)** Novel	*Rhizophora mucronata* (Inner twigs)	*Eurotium chevalieri* KUFA 0006	Eastern Seaboard of Thailand	Antibacterial: MIC (*S. aureus* ATCC 25923) 93.5 μM, MIC (*Enterococcus faecalis* ATCC 29212) 187.0 μM.	[51]
Phomopsanthraquinone **(15)** Novel	*Rhizophora apiculate* (Leaves)	*Phomopsis* sp. PSU- MA214	Songkhla province, Thailand	Cytotoxicity: IC_{50} (MCF-7 cells) 81.3 μM.	[52]
Paradictyoarthrin A **(16)** Novel	Mangrove (Wood)	*Paradictyoarthrinium diffractum* BCC 8704	Laem Son National Park, Ranong Province, Thailand	Cytotoxicity: IC_{50} (KB, MCF-7, Vero and NCI-H187 cell lines) 61.4–82.7 μM.	[53]
Paradictyoarthrin B **(17)** Novel	Mangrove (Wood)	*Paradictyoarthrinium diffractum* BCC 8704	Laem Son National Park, Ranong Province, Thailand	Cytotoxicity: IC_{50} (KB, MCF-7, Vero and NCI-H187 cell lines) 9.2–28.3 μM.	[53]
Tetrahydroaltersolanols C	*Rhizophora apiculate* (Leaves)	*Phomopsis* sp. PSU-MA214	Songkhla province, Thailand	Antibacterial: MIC (*E. coli*) 9.8 μM.	[52]

**(18)**					
Ampelanol **(19)**	*Rhizophora apiculata* (Leaves)	*Phomopsis* sp. PSU-MA214	Songkhla province, Thailand	Antibacterial: MIC (*E. coli*) 7.3 μM.	[52]
Tetrahydroaltersolanol B **(20)**	*Rhizophora apiculata* (Leaves)	*Phomopsis* sp. PSU-MA214	Songkhla province, Thailand	Antifungal: MIC (*Penicillium italicum*) 259.5 μM.	[52]
Altersolanol Q **(21)** Novel	*Avicennia marina*	*Stemphylium globuliferum*	Hurghada, Red Sea, Egypt		[52]
10-methylaltersolanol Q **(22)** Novel	*Avicennia marina*	*Stemphylium globuliferum*	Hurghada, Red Sea, Egypt		[52]
Dihydroaltersolanol B **(23)**	*Avicennia marina*	*Stemphylium globuliferum*	Hurghada, Red Sea, Egypt	Cytotoxicity: IC_{50} (L5178Y mouse lymphoma cell line) 8.4 μM.	[52]
Dihydroaltersolanol C **(24)**	*Avicennia marina*	*Stemphylium globuliferum*	Hurghada, Red Sea, Egypt	Cytotoxicity: IC_{50} (L5178Y mouse lymphoma cell line) 9.7 μM.	[52]
Altersolanol A **(25)**	*Avicennia marina*	*Stemphylium globuliferum*	Hurghada, Red Sea, Egypt	Cytotoxicity: IC_{50} (L5178Y mouse lymphoma cell line) 5.2 μM.	[52]
Altersolanol B **(26)**	*Avicennia marina*	*Stemphylium globuliferum*	Hurghada, Red Sea, Egypt	Cytotoxicity: IC_{50} (L5178Y mouse lymphoma cell line) 15.3 μM.	[52]
Altersolanol N **(27)**	*Avicennia marina*	*Stemphylium globuliferum*	Hurghada, Red Sea, Egypt	Cytotoxicity: IC_{50} (L5178Y mouse lymphoma cell line) 12.6 μM.	[52]
Astronyquinone **(28)** Novel	*Nypa fruticans*	*Astrosphaeriella nypae* BCC 5335	Samut Prakan Province, Thailand	Cytotoxicity: IC_{50} (Vero cells) 57.6 μM.	[55]
TMC-264 **(29)**	*Xylocarpus granatum* (Leaves)	*Penicillium chermesinum* HLit- ROR2	Mangrove Forest Learning and Development Center 2, Samut Sakhon province, Thailand	Antituberculosis: MIC (*Mycobacterium tuberculosis* H37Ra) 165.4 μM.	[56]
Dioxoauroglaucin **(30)** Novel	*Avicennia marina* (Leaves)	*Aspergillus* sp. AV-2	Hurghada, Red Sea, Egypt	Antiproliferative: IC_{50} (Caco-2 cells) 12.3 μM.	[62]
Altenusin **(31)**	Mangrove (Wood)	*Paradictyoarthrinium*	Laem Son National Park, Ranong Province, Thailand	Cytotoxicity: IC_{50} (NCI-H187 cell line) 172.3 μM.	[53]

		diffractum BCC 8704			
Alterporriol X **(32)** Novel	*Avicennia marina*	*Stemphylium globuliferum*	Hurghada, Red Sea, Egypt		[52]
Alterporriol D **(33)**	*Avicennia marina*	*Stemphylium globuliferum*	Hurghada, Red Sea, Egypt	Cytotoxicity: IC_{50} (KB, MCF-7 Vero cells, NCI-H187 cell line) 6.5–10.2 μM.	[52]
Alterporriol E **(34)**	*Avicennia marina*	*Stemphylium globuliferum*	Hurghada, Red Sea, Egypt	Cytotoxicity: IC_{50} (KB, MCF-7 Vero cells, NCI-H187 cell line) 6.5–10.2 μM.	[52]
Alterporriol R **(35)**	*Avicennia marina*	*Stemphylium globuliferum*	Hurghada, Red Sea, Egypt	Cytotoxicity: IC_{50} (KB, MCF-7 Vero cells, NCI-H187 cell line) 6.5–10.2 μM.	[52]
Alterporriol V **(36)**	*Avicennia marina*	*Stemphylium globuliferum*	Hurghada, Red Sea, Egypt	Cytotoxicity: IC_{50} (KB, MCF-7 Vero cells, NCI-H187 cell line) 6.5–10.2 μM.	[52]
Alterporriol W **(37)**	*Avicennia marina*	*Stemphylium globuliferum*	Hurghada, Red Sea, Egypt	Cytotoxicity: IC_{50} (KB, MCF-7 Vero cells, NCI-H187 cell line) 6.5–10.2 μM.	[52]
Rhytidone A **(38)** Novel	*Azima sarmentosa* (Leaves)	*Rhytidhystero n* sp. AS21B	Samutsakhon Province, Thailand	Cytotoxicity: IC_{50} (MCF-7) 14.47 μM, IC_{50} (CaSki) 21.95 μM.	[43]
	Azima sarmentosa (Leaves)	*Rhytidhystero n* sp. AS21B	Samutsakhon Province, Thailand	Cytotoxicity: IC_{50} (MCF-7) 14.47 μM, IC_{50} (CaSki) 21.95 μM.	[43]
Rhytidone B **(39)** Novel	*Azima sarmentosa* (Leaves)	*Rhytidhystero n* sp. AS21B	Samutsakhon Province, Thailand	Cytotoxicity: IC_{50} (CaSki) 22.81 μM.	[43]
Rhytidone C **(40)** Novel	*Azima sarmentosa* (Leaves)	*Rhytidhystero n* sp. AS21B	Samutsakhon Province, Thailand	Cytotoxicity: IC_{50} (MCF-7) 17.30 μM, IC_{50} (CaSki) 24.44 μM.	[43]
MK3018 **(41)**	*Azima sarmentosa* (Leaves)	*Rhytidhystero n* sp. AS21B	Samutsakhon Province, Thailand	Cytotoxicity: IC_{50} (MCF-7) 14.47 μM, IC_{50} (CaSki) 25.59 μM.	[43]
Palmarumycin CR1 **(42)**	*Azima sarmentosa* (Leaves)	*Rhytidhystero n* sp. AS21B	Samutsakhon Province, Thailand	Cytotoxicity: IC_{50} (Ramos) 23.1 μM, IC_{50} (H1975) 50 μM.	[59]
CJ-12,372 **(43)**	*Azima sarmentosa* (Leaves)	*Rhytidhystero n* sp. AS21B	Samutsakhon Province, Thailand		[43]
4-O-methyl-CJ-12,372 **(44)**	*Azima sarmentosa* (Leaves)	*Rhytidhystero n* sp. AS21B	Samutsakhon Province, Thailand		[43]
4-O-methyl-CJ-12,371 **(45)**	*Azima sarmentosa* (Leaves)	*Rhytidhystero n* sp. AS21B	Samutsakhon Province, Thailand		[43]

Palmarumycins P1 **(46)** Novel	Unidentified mangrove (Wood)	BCC 25093	Hat Khanom, Mu Ko Thale Tai National Park, Surat Thani Province, Thailand		[60]
Palmarumycins P2 **(47)** Novel	Unidentified mangrove (Wood)	BCC 25093	Hat Khanom, Mu Ko Thale Tai National Park, Surat Thani Province, Thailand		[60]
Palmarumycins P3 **(48)** Novel	Unidentified mangrove (Wood)	BCC 25093	Hat Khanom, Mu Ko Thale Tai National Park, Surat Thani Province, Thailand		[60]
Palmarumycins P4 **(49)** Novel	Unidentified mangrove (Wood)	BCC 25093	Hat Khanom, Mu Ko Thale Tai National Park, Surat Thani Province, Thailand		[60]
Palmarumycins P5 **(50)** Novel	Unidentified mangrove (Wood)	BCC 25093	Hat Khanom, Mu Ko Thale Tai National Park, Surat Thani Province, Thailand		[60]
Decaspirones A **(51)**	Unidentified mangrove (Wood)	BCC 25093	Hat Khanom, Mu Ko Thale Tai National Park, Surat Thani Province, Thailand	Antimalarial: IC_{50} (*Plasmodium falciparum* K1) 6.84 μM. Cytotoxicity: IC_{50} (Vero cells) 0.54 μM.	[60]
Decaspirones C **(52)**	Unidentified mangrove (Wood)	BCC 25093	Hat Khanom, Mu Ko Thale Tai	Antimalarial: IC_{50} (*P. falciparum* K1) 6.75 μM. Cytotoxicity: IC_{50} (Vero cells) 2.69 μM.	[60]
Palmarumycin CP3 **(53)**	Unidentified mangrove (Wood)	BCC 25093	Hat Khanom, Mu Ko Thale Tai National Park, Surat Thani Province, Thailand		[60]
Palmarumycin CP17 **(54)**	Unidentified mangrove (Wood)	BCC 25093	Hat Khanom, Mu Ko Thale Tai National Park, Surat Thani Province, Thailand		[60]
Palmarumycin M2 **(55)**	Unidentified mangrove (Wood)	BCC 25093	Hat Khanom, Mu Ko Thale Tai National Park, Surat Thani Province, Thailand		[60]
Diaryl ether **(56)**	Unidentified mangrove (Wood)	BCC 25093	Hat Khanom, Mu Ko Thale Tai National Park, Surat Thani Province, Thailand		[60]
Preussomerin C **(57)**	Mangrove (Wood)	*Paradictyoarthrinium*	Laem Son National Park, Ranong Province, Thailand	Cytotoxicity: IC_{50} (NCI-H187) 53.0 μM, IC_{50} (Vero cells) 90.9 μM.	[53]

		diffractum BCC 8704			
YMF 1029C **(58)**	Mangrove (Wood)	*Paradictyoarthrinium diffractum* BCC 8704	Laem Son National Park, Ranong Province, Thailand	Cytotoxicity: IC_{50} (NCI-H187) 13.1 μM, IC_{50} (KB) 34.0 μM, IC_{50} (MCF-7) 99.4 μM.	[53]
Rhytidenone G **(59)** Novel	*Azima sarmentosa* (Leaves)	*Rhytidhysteron rufulum* AS21B	Samutsakhon province, Thailand	Cytotoxicity: IC_{50} (Ramos) 17.98 μM, IC_{50} (H1975) 7.3 μM.	[58]
Rhytidenone H **(60)** Novel	*Azima sarmentosa* (Leaves)	*Rhytidhysteron rufulum* AS21B	Samutsakhon province, Thailand	Cytotoxicity: IC_{50} (Ramos) 0.018 μM, IC_{50} (H1975) 0.252 μM.	[58]
Deoxypreussomerin B **(61)**	*Azima sarmentosa* (Leaves)	*Rhytidhysteron rufulum* AS21B	Samutsakhon province, Thailand	Cytotoxicity: IC_{50} (Ramos) 17.98 μM, IC_{50} (H1975) 7.3 μM.	[58]
1-oxo-1,4-dihydronaphthalene-4- spiro-2′-naphtho[4′,8′-dioxin] **(62)**	*Azima sarmentosa* (Leaves)	*Rhytidhysteron rufulum* AS21B	Samutsakhon province, Thailand		[58]
Preussomerin EG4 **(63)**	*Azima sarmentosa* (Leaves)	*Rhytidhysteron rufulum* AS21B	Samutsakhon province, Thailand		[58]
CJ-12,371 **(64)**	*Azima sarmentosa* (Leaves)	*Rhytidhysteron rufulum* AS21B	Samutsakhon province, Thailand		[58]
Rhytidenone E **(65)**	*Azima sarmentosa* (Leaves)	*Rhytidhysteron rufulum* AS21B	Samutsakhon province, Thailand		[58]
Rhytidenone E **(65)** Novel	*Azima sarmentosa*	*Rhytidhysteron* sp. AS21B	Samutsakhon province, Thailand		[58]
Rhytidenone F **(66)**	*Azima sarmentosa*	*Rhytidhysteron* sp. AS21B	Samutsakhon province, Thailand	Anti-inflammatory: IC_{50} (J774.A1 macrophage cells) 4.90 μM.	[58]

Palmarumycin C5 **(67)**	*Azima sarmentosa* (Leaves)	*Rhytidhysteron rufulum* AS21B	Samutsakhon province, Thailand	Cytotoxicity: IC_{50} (Ramos) 31.7 μM.	[58]
Rhytidenone A **(68)**	*Azima sarmentosa*	*Rhytidhysteron* sp. AS21B	Samutsakhon province, Thailand	Anti-inflammatory: IC_{50} (J774.A1 macrophage cells) 0.31 μM.	[59]
Rhytidenone B **(69)** Novel	*Azima sarmentosa*	*Rhytidhysteron* sp. AS21B	Samutsakhon province, Thailand	Anti-inflammatory: IC_{50} (J774.A1 macrophage cells) 3.60 μM.	[59]
Rhytidenone C **(70)** Novel	*Azima sarmentosa*	*Rhytidhysteron* sp. AS21B	Samutsakhon province, Thailand	Anti-inflammatory: IC_{50} (J774.A1 macrophage cells) 0.31 μM.	[59]
Rhytidenone D **(71)** Novel	*Azima sarmentosa*	*Rhytidhysteron* sp. AS21B	Samutsakhon province, Thailand	Anti-inflammatory: IC_{50} (J774.A1 macrophage cells) 3.60 μM.	[59]
Allantopyrone E **(72)**	*Avicennia marina*	*Aspergillus versicolor*	Port Safaga, Red Sea Governorate, Egypt	Cytotoxicity: IC_{50} (HeLa cells) 50.97± 1.7 μM.	[62]
Pestalopyrone **(73)** Novel	*Avicennia marina* (Leaves)	*Nigrospora oryzae*	Kupang, East Nusa Tenggara, Indonesia		[63]
Cladobotrin V, 5-hydroxymethyl-4- methoxy-6-(E-propenyl)- 2-pyrone) **(74)**	*Rhizophora mucronata*	*Fusarium* sp. IM-37	Muara Angke, Jakarta, Indonesia		[64]
Allantopyrone A **(75)**	*Rhizophora mucronata*	*Fusarium* sp. IM-37	Muara Angke, Jakarta, Indonesia	Showed dose-dependent restoration of growth under Ca^{2+}-induced stress conditions.	[64]
Islandic acid-II methyl ester **(76)**	*Rhizophora mucronata*	*Fusarium* sp. IM-37	Muara Angke, Jakarta, Indonesia	Cytotoxicity: IC_{50} (HL60 cells) 0.32 μM.	[64]
New α-pyrone derivative of compound 85 **(77)** Novel	*Rhizophora mucronata*	*Fusarium* sp. IM-37	Muara Angke, Jakarta, Indonesia	Cytotoxicity: IC_{50} (HL60 cells) 6.55 μM.	[64]

Emericelactone E **(78)** Novel	*Avicennia marina* (Rhizosphere soil)	*Emericella* sp. SWR1718	Jeddah coastline, Saudi Arabia	Cytotoxicity: IC_{50} (HTB-176) 28.3 μM, IC_{50} (SW-620) 46.4 μM.	[50]
12,14-Dihydroxy-3-methyl-3,4,5,6,7,8,9,10-octahydro-1H benzo[c][1] oxacyclododecin-1-one **(79)**	*Bactrospora myriadea*	*Phanerochaete sordida*	Negombo Lagoon, Sri Lanka	Antioxidant: IC_{50} (ABTS assay) 58.91 μM, Anti-inflammatory: IC_{50} (HRBC membrane stabilization) 254.79 μM, IC_{50} (Tyrosinase inhibition) 17.13×10² μM. Cytotoxicity: IC_{50} (CAL-27) 13.65 μM.	[65]
Astronypyrone **(80)**	*Nypa fruticans*	*Astrosphaeriella nypae* BCC 5335	Samut Prakan Province, Thailand	Antioxidant: IC_{50} (HL60 cells) 59.5 μM. Antibacterial: MIC (*Bacillus subtilis*) 80.1 μM.	[66]
Xestodecalactone A **(81)**	*Nypa fruticans*	*Astrosphaeriella nypae* BCC 5335	Samut Prakan Province, Thailand		[66]
Ent-coryoctalactone B **(82)**	*Nypa fruticans*	*Astrosphaeriella nypae* BCC 5335	Samut Prakan Province, Thailand	Antioxidant: IC_{50} (HL60 cells) 108.5 μM. Cytotoxicity: IC_{50} (Vero cells) 56.4 μM.	[66]
Pestalotiopyrone A **(83)** Novel	*Rhizophora apiculata* (Twigs)	*Pestalotiopsis* sp. PSU-MA92	Trang Province, Thailand	Antibacterial: MIC (*B. subtilis*) 154.2 μM.	[67]
Pestalotiopyrone B **(84)** Novel	*Rhizophora apiculata* (Twigs)	*Pestalotiopsis* sp. PSU-MA92	Trang Province, Thailand	Antibacterial: MIC (*S. aureus*) 197.4 μM.	[67]
Pestalotiopyrone C **(85)** Novel	*Rhizophora apiculata* (Twigs)	*Pestalotiopsis* sp. PSU-MA92	Trang Province, Thailand		[67]
Penicilliumolides A **(86)** Novel	*Xylocarpus granatum* (Leaves)	*Penicillium chermesinum* HLit- ROR2	Mangrove Forest Learning and Development Center 2, Samut Sakhon province, Thailand		[64]

Penicilliumolides B **(87)** Novel	*Xylocarpus granatum* (Leaves)	*Penicillium chermesinum* HLit- ROR2	Mangrove Forest Learning and Development Center 2, Samut Sakhon province, Thailand		[64]
Penicilliumolides C **(88)** Novel	*Xylocarpus granatum* (Leaves)	*Penicillium chermesinum* HLit- ROR2	Mangrove Forest Learning and Development Center 2, Samut		[64]
Penicilliumolide s D **(89)** Novel	*Xylocarpus granatum* (Leaves)	*Penicillium chermesinum* HLit- ROR2	Mangrove Forest Learning and Development Center 2, Samut Sakhon province, Thailand		[64]
Penicilliumolides E **(90)**	*Xylocarpus granatum* (Leaves)	*Penicillium chermesinum* HLit- ROR2	Mangrove Forest Learning and Development Center 2, Samut Sakhon province, Thailand		[64]
Pestalolide **(91)** Novel	*Rhizophora apiculata*	*Pestalotiopsis* sp. PSU-MA69	Sutun province, Thailand	Antifungal: MIC (*Candida albicans* NCPF3153 and *Cryptococcus neoformans* ATCC90112) 652.3 μM.	[68]
(−)-Bipolaride A **(92)** Novel	Mangrove (Wood)	*Lophiostoma bipolare* BCC25910	Haad Wanakorn National Park, Thailand	Antimicrobial: MIC (*B. cereus*) 36.5 μM. Cytotoxicity: IC_{50} (MCF-7) 146.1 μM.	[69]
(−)-Scleroderolide **(93)**	Mangrove (Wood)	*Lophiostoma bipolare* BCC25910	Haad Wanakorn National Park, Thailand	Antimicrobial: MIC (*S. aureus* SG511) 24 μM. Cytotoxicity: IC_{50} (MCF-7) 56.3 μM.	[69]
(−)-Bipolaride B **(94)** Novel	Mangrove (Wood)	*Lophiostoma bipolare* BCC25910	Haad Wanakorn National Park, Thailand	Antimicrobial: MIC (*B. cereus*) 62.4 μM. Cytotoxicity: IC_{50} (MCF-7) 120.6 μM.	[69]
(−)-Bipolaride C **(95)** Novel	Mangrove (Wood)	*Lophiostoma bipolare* BCC25910	Haad Wanakorn National Park, Thailand	Antimicrobial: MIC (*B. cereus*) 31.2 μM. Cytotoxicity: IC_{50} (MCF-7) 124.8 μM.	[69]
(−)-Sclerodin methyl ether **(96)**	Mangrove (Wood)	*Lophiostoma bipolare* BCC25910	Haad Wanakorn National Park, Thailand	Cytotoxicity: IC_{50} (MCF-7) 108.3 μM.	[69]

(−)-Sclerodin **(97)**	Mangrove (Wood)	*Lophiostoma bipolare* BCC25910	Haad Wanakorn National Park, Thailand	Enzyme inhibition: IC_{50} (Human leukocyte elastases) 10.9 μM. Cytotoxicity: IC_{50} (MCF-7) 152.4 μM.	[69]
(−)-Bipolaride E **(98)** Novel	Mangrove (Wood)	*Lophiostoma bipolare* BCC25910	Haad Wanakorn National Park, Thailand	Antimicrobial: MIC (*B. cereus*) 39.8 μM. Cytotoxicity: IC_{50} (MCF-7) 146.1 μM.	[69]
Oxasetin **(99)**	Mangrove (Wood)	*Lophiostoma bipolare* BCC25910	Haad Wanakorn National Park, Thailand		[69]
Mairetolide F **(100)**	Mangrove (Wood)	*Xylariaceae* sp. BCC 60405	Ko Hua Ta Chio, Trat Province, Thailand		[70]
13-hydroxymairetolide F **(101)**	Mangrove (Wood)	*Xylariaceae* sp. BCC 60405	Ko Hua Ta Chio, Trat Province, Thailand		[70]
3α-hydroxymairetolide A **(102)**	Mangrove (Wood)	*Xylariaceae* sp. BCC 60405	Ko Hua Ta Chio, Trat Province, Thailand		[70]
Oxysporone **(103)**	*Heritiera fomes* (leaves)	*Pestalotia* sp.	Sundarbans mangrove forest, Bangladesh	Cytotoxicity: anti-MRSA: MIC (EMRSA-15) 32 μM, MIC (SA-1199B) 32 μM.	[71]
Nidulol **(104)** Novel	*Avicennia marina* (Rhizosphere soil)	Emericella sp. SWR1718	Jeddah coastline, Saudi Arabia	Cytotoxicity: IC_{50} (HTB-176) 18.6 μM, IC_{50} (SW-620) 15.7 μM, IC_{50} (HT-29) 36.9 μM.	[50]
Acremonide **(105)** Novel	*Rhizophora apiculata* (Branch)	*Acremonium* sp. PSU-MA70	Satun Province, Thailand		[72]
Penicilliumolides G **(106)**	*Xylocarpus granatum* (Leaves)	*Penicillium chermesinum* HLit- ROR2	Mangrove Forest Learning and Development Center 2, Samut Sakhon province, Thailand	Cytotoxicity: IC_{50} (HL-60) 0.06 μM, IC_{50} (HuCCA-1) 2.19 μM, IC_{50} (HeLa) 1.87 μM, IC_{50} (T47D) 0.81 μM, IC_{50} (MDA-MB-231) 1.36 μM.	[56]
Hypothemycin **(107)**	Mangrove (Wood)	*Aigialus parvus* BCC 5311	Thailand	Antimalarial: IC_{50} (*P. falciparum* K1) 5.8 μM. Cytotoxicity: IC_{50} (KB) 44.9 μM, IC_{50} (BC-1) 16.4 μM, IC_{50} (Vero) 16.6 μM.	[73]

	Mangrove (Wood)	*Aigialus parvus* BCC 5311	Thailand (BIOTEC Culture Collection)	Antimalarial: IC_{50} (*P. falciparum* K1) 7.4 μM. Cytotoxicity: IC_{50} (NCI-H187) 5.3 μM, IC_{50} (Vero cells) 5.6 μM.	[74]
Aigialomycin A **(108)** Novel	Mangrove (Wood)	*Aigialus parvus* BCC 5311	Thailand	Cytotoxicity: IC_{50} (Vero) 11.4 μM. Antimalarial: IC_{50} (*P. falciparum* K1) > 52.9 μM.	[73]
Aigialomycin F **(109)** Novel	Mangrove (Wood)	*Aigialus parvus* BCC 5311	Thailand (BIOTEC Culture Collection)		[74]
Aigialomycin G **(110)** Novel	Mangrove (Wood)	*Aigialus parvus* BCC 5311	Thailand (BIOTEC Culture Collection)		[74]
7′,8′-dihydroaigialomycin F **(111)** Novel	Mangrove (Wood)	*Aigialus parvus* BCC 5311	Thailand (BIOTEC Culture Collection)		[74]
C-6′ alcohol of aigialomycin G aigialomycin G Novel **(112)**	Mangrove (Wood)	*Aigialus parvus* BCC 5311	Thailand (BIOTEC Culture Collection)		[74]
Aigialomycin B **(113)** Novel	Mangrove (Wood)	*Aigialus parvus* BCC 5311	Thailand		[73]
Rearranged resorcylic acid lactone macrolide of 113 Novel **(114)**	Mangrove (Wood)	*Aigialus parvus* BCC 5311	Thailand (BIOTEC Culture Collection)		[74]
Rearranged resorcylic acid lactone macrolide of 114 **(115)** Novel	Mangrove (Wood)	*Aigialus parvus* BCC 5311	Thailand (BIOTEC Culture Collection)		[74]

Aigialone **(116)** Novel	Mangrove (Wood)	*Aigialus parvus* BCC 5311	Thailand		[75]
	Mangrove (Wood)	*Aigialus parvus* BCC 5311	Thailand (BIOTEC Culture Collection)		[74]
Aigialospirol **(117)** Novel	Mangrove (Wood)	*Aigialus parvus* BCC 5311	Thailand		[75]
Benesudon (118) Novel	Mangrove (Wood)	*Aigialus parvus* BCC 5311	Thailand		[75]
Aigialomycin C **(119)** Novel	Mangrove (Wood)	*Aigialus parvus* BCC 5311	Thailand		[73]
Aigialomycin D **(120)** Novel	Mangrove (Wood)	*Aigialus parvus* BCC 5311	Thailand	Antimalarial: IC_{50} (*P. falciparum* K1) 19.7 μM. Cytotoxicity: IC_{50} (KB) 9.0 μM, IC_{50} (Vero) 5.4 μM.	[73]
Aigialomycin E **(121)** Novel	Mangrove (Wood)	*Aigialus parvus* BCC 5311	Thailand		[73]
Dihydrohypothemycin **(122)**	Mangrove (Wood)	*Aigialus parvus* BCC 5311	Thailand		[73]
7′,8′-dihydroaigialospirol **(123)** Novel	Mangrove (Wood)	*Aigialus parvus* BCC 5311	Thailand (BIOTEC Culture Collection)		[74]
4′-deoxy-7′,8′-dihydroaigialospirol **(124)** Novel	Mangrove (Wood)	*Aigialus parvus* BCC 5311	Thailand (BIOTEC Culture Collection)		[74]
Dimethoxy-O-methylpulvinone **(125)**	*Nypa fruticans*	*Astrosphaeriella nypae* BCC 5335	Samut Prakan Province, Thailand	Antimalarial: IC_{50} (*P. falciparum* K1) 17.7 μM. Cytotoxicity: IC_{50} (Vero cells) 58.6 μM.	[55]

Novel					
Pestalotioprolide B **(126)** Novel	*Nypa fruticans, Rhizophora apiculata*	*Pestalotiopsis* sp. PSU-MA119 and PSU-MA92	Thailand		[67]
Pestalotioprolide A **(127)** Novel	*Nypa fruticans, Rhizophora apiculata*	*Pestalotiopsis* sp. PSU-MA119 and PSU-MA92	Thailand		[67]
Triacetate derivative of pestalotioprolide A **(128)**	*Nypa fruticans, Rhizophora apiculata*	*Pestalotiopsis* sp. PSU-MA119 and PSU-MA92	Thailand		[67]
Diacetate derivative of seiricuprolide **(129)**	*Nypa fruticans, Rhizophora apiculata*	*Pestalotiopsis* sp. PSU-MA119 and PSU-MA92	Thailand		[67]
Acetonides 10 **(130)**	Mangrove (Wood)	*Aigialus parvus* BCC. 5311	Thailand	Cytotoxicity: IC_{50} (KB) 19.7 μM, IC_{50} (BC-1) 53.7 μM.	[73]
Acetonides 13 **(131)**	Mangrove (Wood)	*Aigialus parvus* BCC. 5311	Thailand		[73]
Acetonides 11 **(132)**	Mangrove (Wood)	*Aigialus parvus* BCC. 5311	Thailand		[73]
Acetonides 12 **(133)**	Mangrove (Wood)	*Aigialus parvus* BCC. 5311	Thailand		[73]
Acetonides 14 **(134)**	Mangrove (Wood)	*Aigialus parvus* BCC. 5311	Thailand		[73]
Acetonides15 **(135)**	Mangrove (Wood)	*Aigialus parvus* BCC. 5311	Thailand		[73]

Acetonides16 **(136)** Novel	Mangrove (Wood)	*Aigialus parvus* BCC. 5311	Thailand		[73]
Acetonides17 **(137)**	Mangrove (Wood)	*Aigialus parvus* BCC. 5311	Thailand		[73]
Acetonides 18 **(138)**	Mangrove (Wood)	*Aigialus parvus* BCC. 5311	Thailand		[73]
2,6-Dimethoxyphenol **(139)**	Mangrove (Wood)	*Actinomycete* SMS_SU21	Sundarbans mangrove forest, Bangladesh	Antimicrobial: MIC (*C. albicans*) 324 μM.	[80]
Cosmochlorin A **(140)** Novel	*Sonneratia alba*	*Cosmospora vilior* IM2-155	Pagandaran, West Java, Indonesia	Antimicrobial: MIC (*Trichoderma harzianum*) 42.3 μM, MIC (*Aspergillus clavatus*) 169.3 μM, MIC (*C. albicans*) 338.7 μM. Enzyme inhibition: IC_{50} (GSK-3β) 62.5 μM.	[81]
Cosmochlorin B **(141)** Novel	*Sonneratia alba*	*Cosmospora vilior* IM2-155	Pagandaran, West Java, Indonesia	Antimicrobial: MIC (*C. albicans*) > 338.7 μM. Enzyme inhibition: IC_{50} (GSK-3β) 60.6 μM.	[81]
Cosmochlorin C **(142)** Novel	*Sonneratia alba*	*Cosmospora vilior* IM2-155	Pagandaran, West Java, Indonesia	Antimicrobial: MIC (*T. harzianum*) 48.0 μM.	[81]
Tetrahydroauroglaucin **(143)**	*Hibiscus tiliaceus* (sediment around the roots)	*Penicillium oxalicum* OUCMDZ-5207	PakMeng Beach, Thailand	Cytotoxicity: IC_{50} (A549) 5.67μM, IC_{50} (MCF-7) 5.67μM.	[82]
Flavoglaucin **(144)**	*Hibiscus tiliaceus* (sediment around the roots)	*Penicillium oxalicum* OUCMDZ-5207	PakMeng Beach, Thailand	Cytotoxicity: Inhibition (A549 32%, MCF-7 27%) at10 μM	[82]
Auroglaucin **(145)**	*Hibiscus tiliaceus* (sediment around the roots)	*Penicillium oxalicum* OUCMDZ-5207	PakMeng Beach, Thailand	Cytotoxicity: IC_{50} (A549) 5.67μM, IC_{50} (MCF-7) 5.67μM.	[82]

Phenethyl alcohol hydracrylate **(146)**	*Rhizophora apiculata* (Leaves)	*Phomopsis* sp. PSU- MA214	Songkhla Province, Thailand		[52]
Benzene ethanol **(147)**	*Avicennia marina* (Leaves)	*Xylaria feejeensis* AML-02	Wat Asokarambenzene, Samut Prakan, Thailand		[83]
4-hydroxy benzeneethanol **(148)**	*Avicennia marina* (Leaves)	*Xylaria feejeensis* AML-02	Wat Asokarambenzene, Samut Prakan, Thailand		[83]
Asperpentyn **(149)**	*Rhizophora apiculata* (Leaves)	*Pestalotiopsis* sp. PSU-MA69	Sutun province, Thailand		[68]
(S)-penipratynolene **(150)**	*Rhizophora apiculata* (Leaves)	*Pestalotiopsis* sp. PSU-MA69	Sutun province, Thailand		[68]
Phomonitroester **(151)**	*Rhizophora apiculata* (Leaves)	*Phomopsis* sp. PSU- MA214	Songkhla Province, Thailand	Cytotoxicity: IC_{50} (KB) 179.8 μM. Antibacterial: MIC (*S. aureus* ATCC 25923) 418.1 μM.	[52]
3-hydroxy- 4-(1-oxo- ethane) benzoic acid **(152)** Novel	*Avicennia marina* (Fruit)	*Aspergillus versicolor*	7 K Safaga, Red Sea, Egypt	Cytotoxicity: IC_{50} (HeLa cells) 53.5 μM.	[84]
Nigronapthaphenyl **(153)** Novel	*Bruguiera gymnorrhyza* (Leaves)	*Nigrospora sphaerica*	Attaragoda Wetland, Galle, Sri Lanka	Antibacterial: MIC (*B. subtilis* TISTR 088) 5.3–10.7 μM, (*B. cereus* TISTR 688) 5.3–10.7 μM, (*S. aureus* ATCC 43300) 5.3–10.7 μM, (*E. coli* UBC 8161) 5.3–10.7 μM, (methicillin-resistant *S. aureus* ATCC 33591) 5.3–10.7 μM. Cytotoxicity: IC_{50} (HCT 116) 9.62 ± 0.5 μM. Anti-inflammatory: IC_{50} (inhibition of IL-6 release) 6.2 ± 0.5 μM. Enzyme inhibition: IC_{50} (α-glucosidase inhibitory activity) 6.9 ± 0.5 μM.	[85]
Isosclerone **(154)**	*Bruguiera parviflora* (Branches)	*Xylaria cubensis* PSU-MA34	Surat Thani Province, Thailand		[48]

	Bruguiera gymnorrhiza (Leaves)	*Daldinia eschscholtzii* PSU-STD57	Suratthani Province, Thailand		[86]
Bipolarol A **(155)** Novel	Mangrove (Wood)	*Lophiostoma bipolare* BCC25910	Haad Wanakorn National Park, Thailand		[69]
Bipolarol B **(156)** Novel	Mangrove (Wood)	*Lophiostoma bipolare* BCC25910	Haad Wanakorn National Park, Thailand	Cytotoxicity: IC_{50} (KB) 52.5 μM, IC_{50} (MCF-7) 65.3 μM, IC_{50} (NCI-H187) 48.3 μM.	[69]
Bipolarol C **(157)** Novel	Mangrove (Wood)	*Lophiostoma bipolare* BCC25910	Haad Wanakorn National Park, Thailand	Antimicrobial: MIC (*B. cereus*) 62.4 μM.	[69]
Bipolarol D **(158)** Novel	Mangrove (Wood)	*Lophiostoma bipolare* BCC25910	Haad Wanakorn National Park, Thailand		[69]
Pestalotether A **(159)** Novel	*Rhizophora apiculata* (Leaves)	*Pestalotiopsis* sp. PSU-MA69	Sutun province, Thailand	Antifungal: MIC (*C. neoformans*) 595.0 μM.	[68]
Pestalotether B **(160)** Novel	*Rhizophora apiculata* (Leaves)	*Pestalotiopsis* sp. PSU-MA69	Sutun province, Thailand	Antifungal: MIC (*C. neoformans*) 595.0 μM.	[68]
Pestalotether C **(161)** Novel	*Rhizophora apiculate* (Leaves)	*Pestalotiopsis* sp. PSU-MA69	Sutun province, Thailand		[68]
Pestalotether D **(162)** Novel	*Rhizophora apiculata* (Leaves)	*Pestalotiopsis* sp. PSU-MA69	Sutun province, Thailand		[68]
Pestheic acid **(163)**	*Rhizophora apiculata* (Leaves)	*Pestalotiopsis* sp. PSU-MA69	Sutun province, Thailand		[68]
Isosulochrin dehydrate **(164)**	*Rhizophora apiculata* (Leaves)	*Pestalotiopsis* sp. PSU-MA69	Sutun province, Thailand		[68]

Chloroisosulochrin dehydrate **(165)**	*Rhizophora apiculata* (Leaves)	*Pestalotiopsis* sp. PSU-MA69	Sutun province, Thailand	[68]
Chloroisosulochrin **(166)**	*Rhizophora apiculata* (Leaves)	*Pestalotiopsis* sp. PSU-MA69	Sutun province, Thailand	[68]
Isosulochrin **(167)**	*Rhizophora apiculate* (Leaves)	*Pestalotiopsis* sp. PSU-MA69	Sutun province, Thailand	[68]
Siccayne **(168)**	*Rhizophora apiculata* (Leaves)	*Pestalotiopsis* sp. PSU-MA69	Sutun province, Thailand	[68]
Acremonones A **(169)** Novel	*Rhizophora apiculate* (Branch)	*Acremonium* sp. PSU-MA70	Satun Province, Thailand	[72]
Acremonones B **(170)** Novel	*Rhizophora apiculata* (Branch)	*Acremonium* sp. PSU-MA70	Satun Province, Thailand	[72]
Acremonones C **(171)** Novel	*Rhizophora apiculata* (Branch)	*Acremonium* sp. PSU-MA70	Satun Province, Thailand	[72]
Acremonones D **(172)** Novel	*Rhizophora apiculata* (Branch)	*Acremonium* sp. PSU-MA70	Satun Province, Thailand	[72]
Acremonones E **(173)** Novel	*Rhizophora apiculata* (Branch)	*Acremonium* sp. PSU-MA70	Satun Province, Thailand	[72]
Acremonones F **(174)** Novel	*Rhizophora apiculata* (Branch)	*Acremonium* sp. PSU-MA70	Satun Province, Thailand	[72]
Acremonones G **(175)** Novel	*Rhizophora apiculata* (Branch)	*Acremonium* sp. PSU-MA70	Satun Province, Thailand	[72]

Acremonones H **(176)** Novel	*Rhizophora apiculata* (Branch)	*Acremonium sp. PSU-MA70*	Satun Province, Thailand		[72]
(+)-Brefeldin A **(177)**	*Rhizophora apiculata* (Branch)	*Acremonium* sp. PSU-MA70	Satun Province, Thailand	Antifungal: MIC (*C. albicans*) 114.2 μM.	[72]
(R)-(-)-5-Fraxetin **(178)**	*Bruguiera parviflora* (Branch)	*Xylaria cubensis* PSU-MA34	Surat Thani Province, Thailand		[48]
(R)-(-)-5-methoxycarbonylmellein **(179)**	*Bruguiera parviflora* (Branch)	*Xylaria cubensis* PSU-MA34	Surat Thani Province, Thailand		[48]
(R)-(-)-mellein methyl ether **(180)**	*Bruguiera parviflora* (Branch)	*Xylaria cubensis* PSU-MA34	Surat Thani Province, Thailand		[48]
Fraxetin **(181)**	*Ceriops decandra*	*Aspergillus fumigatus*	Sundarbans mangrove forest, Bangladesh	Antibacterial: MIC (*S. aureus*) 30 × 10^2 μM.	[91]
(3R,4R)-4-Hydroxy-5-methylmellein **(182)**	Mangrove (Wood)	*Xylariaceae* sp. BCC 60405	Ko Hua Ta Chio, Trat Province, Thailand		[70]
Altechromone A **(183)**	*Avicennia marina*	*Stemphylium globuliferum*	Hurghada, Red Sea, Egypt	Cytotoxicity: IC_{50} (L5178Y) 14.5 μM.	[54]
Rhytidchromone A **(184)** Novel	*Bruguiera gymnorrhiza* (Leaves)	*Rhytidhysteron rufulum*	Pak Nam Pran, Prachuab Kiri Khan, Thailand	Cytotoxicity: IC_{50} (Kato-3) 23.3 μM, IC_{50} (MCF-7) 19.3 μM.	[66]
Rhytidchromone B **(185)** Novel	*Bruguiera gymnorrhiza* (Leaves)	*Rhytidhysteron rufulum*	Pak Nam Pran, Prachuab Kiri Khan, Thailand	Cytotoxicity: IC_{50} (Kato-3) 21.4 μM.	[66]
Rhytidchromone C **(186)** Novel	*Bruguiera gymnorrhiza* (Leaves)	*Rhytidhysteron rufulum*	Pak Nam Pran, Prachuab Kiri Khan, Thailand		[66]
Rhytidchromone D **(187)** Novel	*Bruguiera gymnorrhiza* (Leaves)	*Rhytidhysteron rufulum*	Pak Nam Pran, Prachuab Kiri Khan, Thailand	Cytotoxicity: IC_{50} (Kato-3) 16.8 μM.	[66]

Rhytidchromone E **(188)** Novel	*Bruguiera gymnorrhiza* (Leaves)	*Rhytidhysteron rufulum*	Pak Nam Pran, Prachuab Kiri Khan, Thailand	Cytotoxicity: IC_{50} (Kato-3) 16.0 μM, IC_{50} (MCF-7) 17.7 μM.	[66]
Pestalochromone A **(189)** Novel	*Rhizophora apiculata* (Leaves)	*Pestalotiopsis* sp. PSU-MA69	Sutun province, Thailand		[68]
Pestalochromone B **(190)** Novel	*Rhizophora apiculata* (Leaves)	*Pestalotiopsis* sp. PSU-MA69	Sutun province, Thailand		[68]
Pestalochromone C **(191)** Novel	*Rhizophora apiculata* (Leaves)	*Pestalotiopsis* sp. PSU-MA69	Sutun province, Thailand		[68]
2,2-dimethyl-2H-1- chromene-6-carboxylic acid **(192)**	*Rhizophora apiculata* (Leaves)	*Pestalotiopsis* sp. PSU-MA69	Sutun province, Thailand		[68]
5, 6,8-dihydroxy-3-(2S-hydroxypropyl)-7- methyl-1H-isochromen- 1-one **(193)** Novel	*Rhizophora mucronata*	*Eurotium chevalieri* KUFA 0006	Eastern Seaboard of Thailand	Anti-biofilm: Inhibited biofilm formation by *E. coli* ATCC 25922 by 50.6% at 255.7 μM.	[51]
Sonneratinone **(194)** Novel	Mangrove	*Aspergillus niger*	Sundarbans mangrove forest, Bangladesh	Antimicrobial: MIC (*Micrococcus luteus*) 31.1 μM, MIC (*S. aureus*) 31.1 μM, MIC (*P. aeruginosa*) 80 μM, MIC (*C. albicans*) 160.1 μM.	[94]
Deuteromycol A **(195)** Novel	Mangrove (Driftwood)	*Deuteromycete sp. MF003*	Shore of the Red Sea, El Gouna, Egypt		[95]
Deuteromycol B **(196)** Novel	Mangrove (Driftwood)	*Deuteromycete* sp. MF003	Shore of the Red Sea, El Gouna, Egypt		[95]
Pestaloxanthone **(197)** Novel	*Rhizophora apiculata*	*Pestalotiopsis* sp. PSU-MA69	Sutun province, Thailand	Antifungal: MIC (*C. albicans* NCPF3153 and *C. neoformans* ATCC90112) 387.6 μM.	[96]

8-Deoxytrichothecin **(198)**	*Rhizophora apiculata* (Branch)	*Acremonium* sp. PSU-MA70	Satun Province, Thailand	Antifungal: MIC (*C. albicans*) 48.5 μM. MIC (*C. neoformans*) ≥ 387.6 μM.	[72]
Sterigmatocystin **(199)** Novel	*Avicennia marina* (Leaves)	*Nigrospora oryzae*	Kupang, East Nusa Tenggara, Indonesia	Cytotoxicity: IC_{50} (L5178Y cell line) 30.8 μM.	[98]
Flavodonfuran **(200)** Novel	*Rhizophora apiculata* (Leaves)	*Flavodon flavus* PSU-MA201	Thailand		[99]
12-methyltetradecanoic acid **(201)**	Mangrove sediments	*Streptomyces albus* MAB56	Andaman Islands, India	Antibacterial: MIC (*S. aureus*) 12.9 μM, MIC (*E. coli*) 51.6 μM.	[101]
Palmitic acid **(202)**	Mangrove sediments	*Streptomyces albus* MAB56	Andaman Islands, India	Antibacterial: MIC (*S. aureus*) 24.4 μM, MIC (*E. coli*) 97.5 μM. Antiviral: IC_{50} (HIV) < 3.9 μM.	[101]
Tridecanoic acid **(203)**	Mangrove sediments	*Streptomyces albus* MAB56	Andaman Islands, India	Antibacterial: MIC (*S. aureus*) 58.3 μM, MIC (*E. coli*) 116.7 μM.	[101]
Xylacinic acids A **(204)** Novel	*Bruguiera parviflora* (Branch)	*Xylaria cubensis* PSU-MA34	Surat Thani Province, Thailand	Cytotoxicity: IC_{50} (KB) 10.7 μM.	[48]
2-hexylidene-3-methylsuccinic acid 4- methyl ester **(205)**	*Bruguiera parviflora* (Branch)	*Xylaria cubensis* PSU-MA34	Surat Thani Province, Thailand	Cytotoxicity: IC_{50} (KB) 13.8 μM.	[48]
Xylacinic acids B **(206)** Novel	*Bruguiera parviflora* (Branch)	*Xylaria cubensis* PSU-MA34	Surat Thani Province, Thailand		[48]
Butanoic acid **(207)**	*Avicennia marina* (Leaves)	*Xylaria feejeensis* AML-02,	Wat Asokaram, Samut Prakan, Thailand		[83]
Kojic acid (1,5-hydroxy- 2-hydroxymethyl-γ- pyrone) **(208)**	*Sonneratia apetala* (Leaves)	*Colletotrichum gloeosporioides*	Sundarbans mangrove forest, Bangladesh	Antibacterial: MIC (*M. luteus*) 7.03×10^2 μM, MIC (*P. aeruginosa*) 8.79×10^2 μM.	[102]

(7R,8R)-8-hydroxysydowic acid **(209)** Novel	*Avicennia marina* (Fruit)	*Aspergillus versicolor*	7 K Safaga, Red Sea, Egypt	Cytotoxicity: IC_{50} (HeLa cells) 43.7 µM.	[84]
(7S,10S)-10-hydroxy-sydowic acid **(210)** Novel	*Avicennia marina* (Fruit)	*Aspergillus versicolor*	7 K Safaga, Red Sea, Egypt		[84]
(7S,11R)-12-hydroxy-sydowic acid **(211)** Novel	*Avicennia marina* (Fruit)	*Aspergillus versicolor*	7 K Safaga, Red Sea, Egypt	Cytotoxicity: IC_{50} (HeLa cells) 83.8 µM.	[84]
(7S,11R)-12-acetoxy- sydowic acid **(212)** Novel	*Avicennia marina* (Fruit)	*Aspergillus versicolor*	7 K Safaga, Red Sea, Egypt		[84]
7-deoxy-7,14-didehydro- 11-hydroxysydonic acid **(213)** Novel	*Avicennia marina* (Fruit)	*Aspergillus versicolor*	7 K Safaga, Red Sea, Egypt	Cytotoxicity: IC_{50} (HeLa cells) 83.8 µM.	[84]
7-deoxy-7,14-didehydro- 12-acetoxy-sydonic acid **(214)** Novel	*Avicennia marina* (Fruit)	*Aspergillus versicolor*	7 K Safaga, Red Sea, Egypt	Cytotoxicity: IC_{50} (HeLa cells) 83.8 µM.	[84]
(E)-7-deoxy-7,8-didehydro-12-acetoxy- sydonic acid **(215)** Novel	*Avicennia marina* (Fruit)	*Aspergillus versicolor*	7 K Safaga, Red Sea, Egypt	Cytotoxicity: IC_{50} (HeLa cells) 53.5 µM.	[84]
(7R,8R)-1,8-epoxy-11-	*Avicennia marina* (Fruit)	*Aspergillus versicolor*	7 K Safaga, Red Sea, Egypt		[84]

hydroxy-sydonic acid **(216)** Novel					
(7R)-11-hydroxy-sydonic acid methyl ester **(217)**	*Avicennia marina* (Fruit)	*Aspergillus versicolor*	7 K Safaga, Red Sea, Egypt		[84]
Taxol **(218)**	*Rhizophora annamalayana* (Leaves)	*Fusarium oxysporum*	Vellar Estuary, Tamil Nadu, India	Cytotoxicity: IC_{50} (KB and KBV200 cells) < 58.6 μM.	[108]
Acaciicolide A, identified as (5aR,9aR)- 3-(hydroxymethyl)- 6,6,9a-trimethyl-6,7,8,9a-tetrahydro-2H-2,5a-methanobenzo[b]oxepin- 9(5H)-one **(219)** Novel	*Bruguiera gymnorrhiza* (Roots)	*Pseudolagarobasidium acaciicola*	Mangrove Forest Learning and Development Center 2, Samut Sakhon province, Thailand		[109]
Acaciicolide B **(220)** Novel	*Bruguiera gymnorrhiza* (Roots)	*Pseudolagarobasidium acaciicola*	Mangrove Forest Learning and Development Center 2, Samut Sakhon province, Thailand		[109]
Acaciicolide C **(221)** Novel	*Bruguiera gymnorrhiza* (Roots)	*Pseudolagarobasidium acaciicola*	Mangrove Forest Learning and Development Center 2, Samut Sakhon province, Thailand		[109]
Acaciicolinol A **(222)** Novel	*Bruguiera gymnorrhiza* (Roots)	*Pseudolagarobasidium acaciicola*	Mangrove Forest Learning and Development Center 2, Samut Sakhon province, Thailand		[109]

Acaciicolinol B **(223)** Novel	*Bruguiera gymnorrhiza* (Roots)	*Pseudolagarobasidium acaciicola*	Mangrove Forest Learning and Development Center 2, Samut Sakhon province, Thailand	Cytotoxicity: IC_{50} (MOLT-3) 165.04 µM, IC_{50} (HL-60) 159.05 µM.	[109]
Acaciicolinol C **(224)** Novel	*Bruguiera gymnorrhiza* (Roots)	*Pseudolagarobasidium acaciicola*	Mangrove Forest Learning and Development Center 2, Samut Sakhon province, Thailand		[109]
Acaciicolinol C **(224)**	*Xylocarpus granatum* (Leaves)	Endophytic fungus XG8D	Samutsakorn province, Thailand	Cytotoxicity: (MCF-7, Hep-G2, KATO-3) at 50 µM.	[110]
Acaciicolinol D **(225)** Novel	*Bruguiera gymnorrhiza* (Roots)	*Pseudolagarobasidium acaciicola*	Mangrove Forest Learning and Development Center 2, Samut Sakhon province, Thailand		[109]
Acaciicolinol D **(225)**	*Xylocarpus granatum* (Leaves)	Endophytic fungus XG8D	Samutsakorn province, Thailand	Cytotoxicity: (MCF-7, Hep-G2, KATO-3) at 50 µM.	[110]
Acaciicolinol E **(226)** Novel	*Bruguiera gymnorrhiza* (Roots)	*Pseudolagarobasidium acaciicola*	Mangrove Forest Learning and Development Center 2, Samut Sakhon province, Thailand		[109]
Acaciicolinol F **(227)** Novel	*Bruguiera gymnorrhiza* (Roots)	*Pseudolagarobasidium acaciicola*	Mangrove Forest Learning and Development Center 2, Samut		[109]
Acaciicolinol F **(227)**	*Xylocarpus granatum* (Leaves)	Endophytic fungus XG8D	Samutsakorn province, Thailand	Cytotoxicity: (MCF-7, Hep-G2, KATO-3) at 50 µM.	[110]
Acaciicolinol G **(228)** Novel	*Bruguiera gymnorrhiza* (Roots)	*Pseudolagarobasidium acaciicola*	Mangrove Forest Learning and Development Center 2, Samut Sakhon province, Thailand		[109]
Acaciicolinol H **(229)** Novel	*Bruguiera gymnorrhiza* (Roots)	*Pseudolagarobasidium acaciicola*	Mangrove Forest Learning and Development Center 2,		[109]

			Samut Sakhon province, Thailand		
Acaciicolinol I **(230)** Novel	*Bruguiera gymnorrhiza* (Roots)	*Pseudolagarobasidium acaciicola*	Mangrove Forest Learning and Development Center 2, Samut Sakhon province, Thailand		[109]
Acaciicolinol J **(231)** Novel	*Bruguiera gymnorrhiza* (Roots)	*Pseudolagarobasidium acaciicola*	Mangrove Forest Learning and Development Center 2, Samut Sakhon province, Thailand		[109]
Acaciicolinol K **(232)** Novel	*Bruguiera gymnorrhiza* (Roots)	*Pseudolagarobasidium acaciicola*	Mangrove Forest Learning and Development Center 2, Samut		[109]
Acaciicolinol K **(232)**	*Xylocarpus granatum* (Leaves)	Endophytic fungus XG8D	Samutsakorn province, Thailand	Cytotoxicity: (MCF-7, Hep-G2, KATO-3) at 50 μM.	[110]
Acaciicolinol L **(233)** Novel	*Bruguiera gymnorrhiza* (Roots)	*Pseudolagarobasidium acaciicola*	Mangrove Forest Learning and Development Center 2, Samut Sakhon province, Thailand		[109]
Spiroacaciicolide B **(234)** Novel	*Bruguiera gymnorrhiza* (Roots)	*Pseudolagarobasidium acaciicola*	Mangrove Forest Learning and Development Center 2, Samut Sakhon province, Thailand		[109]
Spiroacaciicolide C **(235)** Novel	*Bruguiera gymnorrhiza* (Roots)	*Pseudolagarobasidium acaciicola*	Mangrove Forest Learning and Development Center 2, Samut Sakhon province, Thailand		[109]
7-epi-merulin B **(236)** Novel	*Bruguiera gymnorrhiza* (Roots)	*Pseudolagarobasidium acaciicola*	Mangrove Forest Learning and Development Center 2, Samut Sakhon province, Thailand	Cytotoxicity: IC_{50} (HL-60) 0.28 μM.	[109]
3-epi-merulin A **(237)** Novel	*Bruguiera gymnorrhiza* (Roots)	*Pseudolagarobasidium acaciicola*	Mangrove Forest Learning and Development Center 2, Samut	Cytotoxicity: IC_{50} (cancer cell lines) 12.09–170.08 μM.	[109]

(3S,4R,6aS,10aR) -4- hydroxy-7,7,10a-trimethyl-5,6,7,10a-tetrahydro-3H-3,6a-methanobenzo[c][1,2] dioxocin-10(4H)-one Novel **(238)**	*Bruguiera gymnorrhiza* (Roots)	*Pseudolagarobasidium acaciicola*	Mangrove Forest Learning and Development Center 2, Samut Sakhon province, Thailand		[109]
Merulin A **(239)**	*Bruguiera gymnorrhiza* (Roots)	*Pseudolagarobasidium acaciicola*	Mangrove Forest Learning and Development Center 2, Samut Sakhon province, Thailand		[109]
Merulin A **(239)** Novel	*Xylocarpus granatum*	*Basidiomycetous* fungus XG8D	Samutsakorn Province, Thailand	Cytotoxicity: IC_{50} (BT474) 19.0 μM, IC_{50} (SW620) 19.6 μM.	[111]
Merulin A **(239)**	*Xylocarpus granatum*	*Basidiomycetous* fungus XG8D	Samutsakorn Province, Thailand		[112]
Merulin D **(240)**	*Bruguiera gymnorrhiza* (Roots)	*Pseudolagarobasidium acaciicola*	Mangrove Forest Learning and Development Center 2, Samut Sakhon province, Thailand		[109]
Merulin D **(240)** Novel	*Xylocarpus granatum*	*Basidiomycetous* fungus XG8D	Samutsakorn Province, Thailand		[112]
Merulinol A **(241)** Novel	*Bruguiera gymnorrhiza* (Roots)	*Pseudolagarobasidium acaciicola*	Mangrove Forest Learning and Development Center 2, Samut		[110]
Merulinol B **(242)** Novel	*Bruguiera gymnorrhiza* (Roots)	*Pseudolagarobasidium acaciicola*	Mangrove Forest Learning and Development Center 2, Samut Sakhon province, Thailand		[110]

Merulinol C **(243)** Novel	*Bruguiera gymnorrhiza* (Roots)	*Pseudolagarobasidium acaciicola*	Mangrove Forest Learning and Development Center 2, Samut Sakhon province, Thailand	Cytotoxicity: IC_{50} (KATO-3) 35.0 μM.	[110]
Merulinol D **(244)** Novel	*Bruguiera gymnorrhiza* (Roots)	*Pseudolagarobasidium acaciicola*	Mangrove Forest Learning and Development Center 2, Samut Sakhon province, Thailand	Cytotoxicity: IC_{50} (KATO-3) 25.3 μM.	[110]
Merulinol E **(245)** Novel	*Bruguiera gymnorrhiza* (Roots)	*Pseudolagarobasidium acaciicola*	Mangrove Forest Learning and Development Center 2, Samut Sakhon province, Thailand		[110]
Merulinol F **(246)** Novel	*Bruguiera gymnorrhiza* (Roots)	*Pseudolagarobasidium acaciicola*	Mangrove Forest Learning and Development Center 2, Samut Sakhon province, Thailand		[110]
PR-Toxin **(247)**	*Xylocarpus granatum* (Leaves)	*Penicillium chermesinum* HLit-ROR2	Mangrove Forest Learning and Development Center 2, Samut Sakhon province, Thailand	Cytotoxicity: (T47D, MDA-MB-231, HepG2, and MOLT-3 cell lines).	[56]
Penicilliumolides F **(248)**	*Xylocarpus granatum* (Leaves)	*Penicillium chermesinum* HLit- ROR2	Mangrove Forest Learning and Development Center 2, Samut Sakhon province, Thailand	Cytotoxicity: (T47D, MDA-MB-231, HepG2, and MOLT-3 cell lines).	[56]
Seiridin **(249)**	*Rhizophora apiculata*	*Pestalotiopsis* sp. PSU-MA69	Sutun province, Thailand		[68]
Tremulenolide A **(250)**	*Rhizophora apiculata* (Leaves)	*Flavodon flavus* PSU-MA201	Thailand	Antimicrobial: MIC (*S. aureus* ATCC 25923) 484.3 μM, MIC (*C. neoformans* ATCC 90113) 484.3 μM.	[99]
Ampelanol **(251)**	*Rhizophora apiculata* (Leaves)	*Phomopsis* sp. PSU- MA214	Songkhla Province, Thailand	Cytotoxicity: IC_{50} (MCF-7) > 146.9 μM.	[52]
Merulin B Novel **(252)**	*Xylocarpus granatum*	*Basidiomycetous* fungus XG8D	Samutsakorn Province, Thailand		[111]

Merulin B **(252)**	*Xylocarpus granatum*	Endophytic fungus XG8D	Samutsakorn Province, Thailand		[112]
	Bruguiera gymnorrhiza	*Pseudolagarobasidium acaciicola*	Thailand	Cytotoxicity: IC_{50} (MOLT-3) 44.2 μM, IC_{50} (HepG2) 181.6 μM.	[113]
Merulin C Novel **(253)**	*Xylocarpus granatum*	*Basidiomycetous* fungus XG8D	Samutsakorn Province, Thailand	Cytotoxicity: IC_{50} (BT474) 5.5 μM, IC_{50} (SW620) 14.5 μM.	[111]
Merulin C **(253)**	*Xylocarpus granatum*	Endophytic fungus XG8D	Samutsakorn Province, Thailand	Antiangiogenic: IC_{50} (micro vessel sprouting) 2.5 μM, IC_{50} (HUVEC proliferation) IC_{50} 0.9 μM.	[112]
	Bruguiera gymnorrhiza	*Pseudolagarobasidium acaciicola*	Thailand	Cytotoxicity: IC_{50} (MB-231) 0.28 μM, IC_{50} (HL-60) 13.18 μM.	[113]
Steperoxide A **(254)**	*Xylocarpus granatum*	Endophytic fungus XG8D	Samutsakorn Province, Thailand	Cytotoxicity: IC_{50} (HuCCA-1) 2.7 μM, IC_{50} (MDA-MB-231) 14.6 μM. Antiangiogenic: Complete inhibition at 25 μM.	[112]
	Bruguiera gymnorrhiza	*Pseudolagarobasidium acaciicola*	Thailand	Cytotoxicity: IC_{50} (MB-231) 2.7 μM, IC_{50} (HL-60) 21.3 μM.	[113]
Acaicolin A **(255)** Novel	*Bruguiera gymnorrhiza*	*Pseudolagarobasidium acaciicola*	Thailand		[113]
Spiroacaciicolide A **(256)** Novel	*Bruguiera gymnorrhiza*	*Pseudolagarobasidium acaciicola*	Thailand		[113]
Spiroacaciicolide A **(256)**	Mangrove (Wood)	*Xylariaceae* sp. BCC 60405	Ko Hua Ta Chio, Trat Province, Thailand		[70]
3-epi-steperoxide A **(257)** Novel	*Bruguiera gymnorrhiza*	*Pseudolagarobasidium acaciicola*	Thailand	Cytotoxicity: IC_{50} (HuCCA-1) 2.7 μM, IC_{50} (MDA-MB-231) 14.6 μM.	[113]
	Mangrove (Wood)	*Xylariaceae* sp. BCC 60405	Ko Hua Ta Chio, Trat Province, Thailand	Cytotoxicity: IC_{50} (Vero cells) 119.1 μM.	[70]
Eremophilanolide A **(258)** Novel	Mangrove (Wood)	*Xylariaceae* sp. BCC 60405	Ko Hua Ta Chio, Trat Province, Thailand		[70]

Eremophilanolide B **(259)** Novel	Mangrove (Wood)	*Xylariaceae* sp. BCC 60405	Ko Hua Ta Chio, Trat Province, Thailand		[70]
Eremophilanolide C **(260)** Novel	Mangrove (Wood)	*Xylariaceae* sp. BCC 60405	Ko Hua Ta Chio, Trat Province, Thailand		[70]
Xylaremophil **(261)**	Mangrove (Wood)	*Xylariaceae* sp. BCC 60405	Ko Hua Ta Chio, Trat Province, Thailand	Antibacterial: MIC (*M. luteus*) 114.5 μM.	[70]
Xylaremophil A **(262)**	Mangrove (Wood)	*Xylariaceae* sp. BCC 60405	Ko Hua Ta Chio, Trat Province, Thailand		[70]
Crotonepoxide **(263)**	Mangrove (Wood)	*Xylariaceae* sp. BCC 60405	Ko Hua Ta Chio, Trat Province, Thailand		[70]
7-hydroxyxylareromophil **(264)**	Mangrove (Wood)	*Xylariaceae* sp. BCC 60405	Ko Hua Ta Chio, Trat Province, Thailand		[70]
13-hydroxy-xylareremophil A **(265)**	Mangrove (Wood)	*Xylariaceae* sp. BCC 60405	Ko Hua Ta Chio, Trat Province, Thailand		[70]
Eremophilane **(266)** Novel	Mangrove (Wood)	*Xylariaceae* sp. BCC 60405	Ko Hua Ta Chio, Trat Province, Thailand		[70]
Bipolarenic acid **(267)** Novel	Mangrove (Wood)	*Lophiostoma bipolare* BCC25910	Haad Wanakorn National Park, Thailand		[69]
7-epi-tessaric acid **(268)** Novel	Mangrove (Wood)	*Xylariaceae* sp. BCC 60405	Ko Hua Ta Chio, Trat Province, Thailand		[70]
Fumigaclavine C **(269)**	*Ceriops decandra* (Leaves)	*Aspergillus fumigatus*	Sundarbans mangrove forest, Bangladesh	Antibacterial: MIC (*P. aeruginosa*) 425.8 μM, MIC (*M. luteus*) 212.9 μM.	[91]
(3R)-5,7-Dihydroxy-3-methylisoindolin-1-one **(270)** Novel	*Avicennia marina*	Unidentified endophytic fungus	Oman	Antibacterial: MIC (*Streptococcus pneumoniae*) 331.3 μM.115	[50]

2-(2-methyl-3-en-2-yl)- 1H-indole-3-carbaldehyde **(271)** Novel	*Rhizophora mucronata*	*Eurotium chevalieri* KUFA 0006	Eastern Seaboard of Thailand	Antibacterial: MIC (*S. aureus* ATCC 25923) 300.1 μM.	[51]
2-(2,2-dimethylcyclopropyl)- 1H-indole-3-carbaldehyde **(272)** Novel	*Rhizophora mucronata*	*Eurotium chevalieri* KUFA 0006	Eastern Seaboard of Thailand		[51]
Anthcolorin G **(273)** Novel	*Avicennia marina* (Fruit)	*Aspergillus versicolor*	Red Sea, Egypt		[84]
Anthcolorin H **(274)** Novel	*Avicennia marina* (Fruit)	*Aspergillus versicolor*	Red Sea, Egypt	Cytotoxicity: IC_{50} (HeLa cells) 99.8 μM.	[84]
Aliphatic amide **(275)**	*Rhizophora apiculata* (Leaves)	*Phomopsis* sp. PSU- MA214	Songkhla Province, Thailand		[52]
Cytochalasin H **(276)** Novel	*Sonneratia griffithii* (Bark)	*Diaporthe amygdali* SgKB4	Bungus Coast, Padang, West Sumatra, Indonesia		[117]
Phomopsichalasin D **(277)** Novel	*Xylocarpus granatum* (Leaves)	*Phomopsis* sp. xy21	Trang Province, Thailand		[118]
Phomopsichalasin E **(278)** Novel	*Xylocarpus granatum* (Leaves)	*Phomopsis* sp. xy21	Trang Province, Thailand	Cytotoxicity: IC_{50} (HCT-8) 42.9 μM, IC_{50} (A2780) 87.4 μM.	[118]
Phomopsichalasin F **(279)** Novel	*Xylocarpus granatum* (Leaves)	*Phomopsis* sp. xy21	Trang Province, Thailand	Cytotoxicity: IC_{50} (HCT-8/T) 11.2 μM, IC_{50} (A2780) 8.6 μM.	[118]
Phomopsichalasin G **(280)**	*Xylocarpus granatum* (Leaves)	*Phomopsis* sp. xy21	Trang Province, Thailand	Cytotoxicity: IC_{50} (HCT-8) 7.5 μM, IC_{50} (A549) 6.4 μM, IC_{50} (MDA-MB-231) 3.4 μM.	[118]

Novel					
Cytochalasin D **(281)**	*Bruguiera parviflora* (Branch)	*Xylaria cubensis* PSU-MA34	Surat Thani Province, Thailand	Cytotoxicity: IC_{50} (KB cells) 7.9 μM.	[48]
(3,1′-Didehydro-3-[2″- (3″,3″′-dimethyl-prop-2-enyl)-3″′-indolylmethylene]-6-methylpiperazine-2,5- dione) **(282)** Novel	*Porteresia coarctata*	*Penicillium chrysogenum* MTCC 5108	Goa, India	Antibacterial: MIC (*V. cholerae*) 49.5 μM.	[119]
(11S, 14R)-cyclo(tryptophyl valyl) **(283)**	*Rhizophora mucronata*	*Eurotium chevalieri* KUFA 0006	Eastern Seaboard of Thailand		[51]
Echinulin **(284)**	*Rhizophora mucronata*	*Eurotium chevalieri* KUFA 0006	Eastern Seaboard of Thailand		[51]
Eurocristatine **(285)**	*Rhizophora mucronata*	*Eurotium chevalieri* KUFA 0006	Eastern Seaboard of Thailand		[51]
Azaspirofuran B **(286)**	*Ceriops decandra*	*Aspergillus fumigatus*	Sundarbans mangrove forest, Bangladesh	Antibacterial: MIC (*S. aureus*) 96.2 μM.	[91]
Adenine **(287)**	*Avicennia marina*	*Emericella* sp. SWR1718	Jeddah coastline, Saudi Arabia		[50]
Adenosine **(288)**	*Avicennia marina*	*Emericella* sp. SWR1718	Jeddah coastline, Saudi Arabia	Cytotoxicity: IC_{50} (HTB-176) 42.9 μM.	[50]
3'-deoxyadenosine **(289)**	*Avicennia marina*	*Emericella* sp. SWR1718	Jeddah coastline, Saudi Arabia	Cytotoxicity: IC_{50} (HTB-176) 40.7 μM, IC_{50} (SW-620) 77.8 μM, IC_{50} (HT-29) 75.2 μM.	[50]
3'-deoxy-5'-acetyladenosine **(290)**	*Avicennia marina*	*Emericella* sp. SWR1718	Jeddah coastline, Saudi Arabia		[50]

1-(2′,6′-Dimethylphenyl) - 2-n-propyl-1,2-dihydropyridazine-3,6- dione **(291)** Novel	*Avicennia marina* (Leaves)	*Aspergillus* sp. AV-2	Hurghada, Red Sea, Egypt	Cytotoxicity: IC_{50} (Caco-2 cells) 18.2 μM.	[62]
Astronyurea **(292)** Novel	*Nypa fruticans*	*Astrosphaeriella nypae* BCC 5335	Samut Prakan Province, Thailand	Antibacterial: MIC (*S. aureus*) 159.8 μM, MIC (*E. coli*) 228.2 μM. Cytotoxicity: IC_{50} (Vero cells) 105.9 μM.	[55]
2-[(2,2-dimethylbut-3-enoyl) amino] benzoic acid **(293)** Novel	*Rhizophora mucronata*	*Eurotium chevalieri* KUFA 0006	Eastern Seaboard of Thailand	Antibacterial: MIC (*Enterococcus faecalis* ATCC 29212) 274.4 μM.	[51]
1,2,4-Triazolo [1,5-a] pyrimidine, 5,7-dimethyl-2-phenyl **(294)**	Mangrove	*Actinomycetes* SMS_SU21	Sundarbans mangrove forest, Bangladesh	Antifungal: MIC (*Rhizoctonia solani*) 209.8 μM, MIC (*Macrophomina phaseolina*) 209.8 μM. Antibacterial: MIC (*E. coli*) 210 μM.	[80]
Vinylbital **(295)**	Mangrove (Wood)	*Actinomycete strain* SMS_SU21	Sundarbans mangrove forest, Bangladesh	Antibacterial: MIC (*V. cholerae*) 167.9 μM, MIC (*S. aureus*) 167.9 μM.	[80]
Farinomalein A **(296)**	*Avicennia marina* (Leaves)	Unidentified endophytic fungus	Oman		[112]
Farinomalein B **(297)**	*Avicennia marina* (Leaves)	Unidentified endophytic fungus	Oman	Cytotoxicity: IC_{50} (Lymphoma cell line L5178Y) 20.8 μM.	[112]
Farinomalein C **(298)** Novel	*Avicennia marina* (Leaves)	Unidentified endophytic fungus	Oman		[112]

Farinomalein D **(299)** Novel	*Avicennia marina* (Leaves)	Unidentified endophytic fungus	Oman		[112]
Farinomalein E **(300)** Novel	*Avicennia marina* (Leaves)	Unidentified endophytic fungus	Oman		[112]
Guanomide A **(301)**	*Rhizophora apiculata* (Branch)	*Acremonium* sp. PSU-MA70	Satun Province, Thailand		[72]
Guangomide B **(302)**	*Rhizophora apiculata* (Branch)	*Acremonium* sp. PSU-MA70	Satun Province, Thailand		[72]
